# NLRP3 Inflammasome Mediates Dormant Neutrophil Recruitment following Sterile Lung Injury and Protects against Subsequent Bacterial Pneumonia in Mice

**DOI:** 10.3389/fimmu.2017.01337

**Published:** 2017-10-31

**Authors:** Xiaoli Tian, He Sun, Amy-Jo Casbon, Edward Lim, Kevin P. Francis, Judith Hellman, Arun Prakash

**Affiliations:** ^1^Department of Anesthesia and Perioperative Care, University of California, San Francisco, San Francisco, CA, United States; ^2^Department of Anatomy, University of California, San Francisco, San Francisco, CA, United States; ^3^Preclinical Imaging, PerkinElmer, Hopkinton, MA, United States; ^4^Division of Critical Care Medicine, Department of Anthesia and Perioperative Care, University of California, San Francisco, San Francisco, CA, United States

**Keywords:** lung injury, inflammasome, NOD-, LRR-, and pyrin domain-containing 3, interleukin-1β, ischemia–reperfusion, inflammation, neutrophil activation

## Abstract

Sterile lung injury is an important clinical problem that complicates the course of severely ill patients. Interruption of blood flow, namely ischemia–reperfusion (IR), initiates a sterile inflammatory response in the lung that is believed to be maladaptive. The rationale for this study was to elucidate the molecular basis for lung IR inflammation and whether it is maladaptive or beneficial. Using a mouse model of lung IR, we demonstrate that sequential blocking of inflammasomes [specifically, NOD-, LRR-, and pyrin domain-containing 3 (NLRP3)], inflammatory caspases, and interleukin (IL)-1β, all resulted in an attenuated inflammatory response. IL-1β production appeared to predominantly originate in conjunction with alveolar type 2 epithelial cells. Lung IR injury recruited unactivated or dormant neutrophils producing less reactive oxygen species thereby challenging the notion that recruited neutrophils are terminally activated. However, lung IR inflammation was able to limit or reduce the bacterial burden from subsequent experimentally induced pneumonia. Notably, inflammasome-deficient mice were unable to alter this bacterial burden following IR. Thus, we conclude that the NLRP3 inflammasome, through IL-1β production, regulates lung IR inflammation, which includes recruitment of dormant neutrophils. The sterile IR inflammatory response appears to serve an important function in inducing resistance to subsequent bacterial pneumonia and may constitute a critical part of early host responses to infection in trauma.

## Introduction

Reperfusion injury following ischemia is believed to contribute to single and multiorgan failure and mortality in septic and severely injured trauma patients [reviewed in Ref. (1)]. Significant progress has been made over the past decade in understanding how the immune system responds to infection and injury, and several mechanistic pathways have been identified downstream of sterile injury, including downstream of ischemia–reperfusion (IR) injury (2–4). Yet, in the lung, the key mechanisms underpinning IR injury are poorly understood and the sterile inflammation that accompanies this type of injury is thought to be detrimental (5). Understanding the mechanisms underlying IR injury in the lungs is important given the vital physiological role this organ system plays, and its position at an interface between the host and environment. As such, the respiratory system is extremely susceptible to injury with even temporary functional impairment threatening the life of the individual. By elucidating key innate immune pathways that control and regulate lung IR inflammation, this response could potentially be harnessed or manipulated to protect or enhance lung function in various clinical contexts arising from sterile injuries or exposure to pathogen.

While studies on sterile lung injury are numerous in the literature, few focus on the topic of lung IR injury, and even fewer on ventilated lung IR injury, i.e., IR without hypoxia. Direct or indirect lung IR injury is encountered during lung transplantation, hemorrhagic trauma, pulmonary emboli, and even low flow states, such as sepsis and these conditions may or may not involve tissue hypoxia. The lung is a large portal for entry of foreign material and microorganisms and is susceptible to many sterile and non-sterile challenges. IR can contribute to acute lung injury (ALI) in trauma (6). Studies involving animal models of IR injury in various organs, including heart, kidney, liver, GI tract, lung, and brain have demonstrated that IR can trigger-specific signaling pathways and results in damaging inflammation (7–21). However, detailed mechanisms and consequences of IR inflammation in the ventilated lung especially in the context of infection have not been well studied. Whether or not a beneficial role exists for this type of inflammation is unknown.

Molecular pathways that have been identified to contribute to sterile inflammation in general and to that from generalized organ IR injury in particular include toll-like receptor (TLR) pathways, specific inflammasome complexes, and IL-1 signaling pathways [(22) and reviewed in Ref. (4)]. TLR4 has been shown by us, and others, to be an important mediator of the inflammatory response in ventilated and non-ventilated lung IR injury (23–25). Additionally, IL-1β has been detected locally in the lung and in circulating plasma following ventilated lung IR injury (25). Upstream of IL-1β, the inflammasome has been shown to be important in the context of myocardial and renal IR injury, and in lung transplantation, but not in conditions of *in vivo* lung IR injury (14, 15, 26). The inflammasome refers to a family of macromolecular protein complexes that coordinate IL-1β release through a two-step mechanism in which first, priming occurs resulting in pro-IL-1β protein being made and second, processing of IL-1β into its mature form occurs through the inflammasome’s activation of inflammatory caspases. All inflammasome family members contain adaptor and effector subunits with the ASC adaptor protein being a common structural component of all types of assembled inflammasomes. Among the various inflammasome complexes identified thus far, the NOD-, LRR-, and pyrin domain-containing 3 (NLRP3) inflammasome is considered to be an integrator of a diverse sterile and some non-sterile injury signals as well as the primary regulator of the resultant immune response (4, 14, 27–29). However, most studies that have implicated specific molecules or pathways in IR have focused on upstream sensing of injury (including the sensing of HMGB1, histones, ATP, uric acid, formyl peptides, nucleic acids, reactive oxygen species, mechanical stress, etc.) or on the late phase of neutrophil recruitment and organ damage. Interestingly, the long-held belief that neutrophils recruited to tissue injury are terminally activated to fulfill their roles of pathogen defense, bystander tissue damage, and/or tissue repair has recently come under challenge (30). There is emerging evidence that neutrophils may exist in various subtypes, have multifaceted roles, and possess intermediate stages of activation even after leaving the circulation [(31), reviewed in Ref. (32, 33)]. To the best of our knowledge the activation state and function of recruited neutrophils in lung IR injury has not been studied.

*In vivo* lung IR is challenging to study in preclinical small animal models. We employ a microsurgical lung IR injury model in mice, which selectively interrupts blood supply to the left lung without affecting ventilation and without accompanying tissue hypoxia. This model allows us to precisely examine the isolated process of lung IR *in vivo* and with it we have identified TLR4 and alveolar macrophages (AMs) as important contributors to the lung IR inflammatory process and our *in vitro* studies have suggested that IL-1β may also play a key role (25, 34). We hypothesized that lung IR injury may trigger inflammasome-mediated production of IL-1β, a powerful initiator cytokine produced early after IR and that it would originate from or in close proximity to specific IR injury sensing cell type(s) in the lung. We further hypothesized, based on our prior work demonstrating the transient nature of lung IR inflammation (34), that this inflammation may not *per se* result in long-term lung dysfunction, but instead may assist in the clearance of infections that often accompany traumatic injuries.

In this study, we demonstrated that lung IR injury triggers activation of the NLRP3 inflammasome and release of IL-1β, both of which were required for the lung IR inflammatory response. Additionally, we have identified the alveolar type 2 (AT2) epithelial cell as an important cell involved in the release of IL-1β. Furthermore, lung IR injury initiates cytokine/chemokine cascades that result in the recruitment of dormant neutrophils, which we propose to define as neutrophils that arrive to the site of injury but are not yet triggered for full activation. The benefits of this NLRP3-dependent sterile inflammation were evident only when bacterial infection was introduced at the site of lung IR. Overall, our study reveals a role for IL-1β regulation, through the NLRP3 inflammasome, in initiating lung IR inflammation which includes the recruitment of dormant neutrophils, as well as a beneficial role for this sterile immune response in combating infection.

## Materials and Methods

### Animals

All animal studies were approved by the Institutional Animal Care and Use Committee at the University of California, San Francisco (Protocol# AN152943). Male mice (12–15 weeks old) were either purchased (The Jackson Laboratory, Bar Harbor, ME, USA) or bred at the animal facility at University of California, San Francisco. Purchased mice were allowed to acclimatize to their new housing for at least 1 week before any experiments on them were conducted. ASC and NLRP3 KO mice were a kind gift from V. Dixit (Genentech) and IL-1R and Caspase 1 KO mice were a kind gift from A. Ma (UCSF). All mice were on a C57B/L6 background except for Caspase 1 KO mice which were on a 129 background and thus also Caspase 11 deficient. B6 Albino mice were provided by Kevin P. Francis (Perkin Elmer). tdTomato/MRP8 Cre mice were generously provided by Zena Werb (UCSF).

Only male mice were used in our experiments primarily to reflect the fact that trauma disproportionately affects human males. Based on our previous studies, we used group sizes of 6–10 for all experiments (25, 34). All mice for a given experiment were either littermates or purchased/bred such that they were age matched. Mice used in these experiments were randomly chosen either to undergo the various surgeries (sham vs. IR) or treatments (±specific treatments), therefore there was no attempt made to blind the individuals conducting the experiments, however, in situations where mice received a treatment or control before IR surgery or infection after surgery, the individual collecting the organs/plasma and generating the enzyme-linked immunosorbant assay (ELISA)/qPCR data was unaware of which mice received which specific treatment.

### Ventilated Lung IR [Unilateral Left Pulmonary Artery (PA) Occlusion] Surgery

A murine model of unilateral left PA occlusion was used, as we have described previously (25). Briefly, anesthetized mice [using IP tribromoethanol (Avertin^®^); Sigma-Aldrich] were orally intubated, given buprenorphine (IP; Harry Schein, Melville, NY, USA), and placed on a rodent ventilator, using tidal volumes of 225 µL (7.5 cm^3^/kg), and a respiratory rate of 180 breaths/min (assuming an average mouse weight of 30 g). A left thoracotomy *via* the interspace between the second and third ribs was performed and the left PA was identified and ligated using a slip knot suture with 7-0 or 8-0 prolene monofilament suture. The end of the suture was externalized through a narrow bore (27 g) needle to the anterior chest wall. Prior to closure of the thorax, the left lung was reinflated with positive end pressure. Local anesthetic (3–4 drops of 0.25% bupivacaine) was applied topically prior to skin closure. The total period of mechanical ventilation and surgery was approximately 20–25 min. After skin closure, mice were extubated and allowed to recover from anesthesia. After 30–60 min of ischemia, the ligature on the PA was released and left lung reperfusion started. At the experimental end point times, mice were euthanized and the blood and lungs were collected.

Blood was collected from anesthetized mice *via* cardiac puncture using a heparinized syringe, centrifuged (14,000*g*, 5 min) and the plasma separated, flash frozen in liquid nitrogen and stored at −80°C. Lower portions of the left lungs were excised and placed in either Trizol^®^ (Life Technologies, Carlsbad, CA, USA) at −80°C for RNA isolation. Levels of cytokines and chemokines were quantified in plasma. Lungs, spleen, liver, and kidneys were collected as well for colony-forming unit (CFU) measurements in infection experiments as described later.

Sham (control) mice underwent left thoracotomy and all other procedures at precisely the same time points as experimental mice, except the left PA was not isolated and the slip-knot was not tied or externalized.

All mice (sham and IR) received equivalent durations of mechanical ventilation (20–25 min) and were left spontaneously breathing during their recovery from anesthesia and the remainder of the ischemia period and subsequent reperfusion or equivalent periods in the sham mice.

While this lung IR procedure has high initial survival rates of 80–90% on average, some mice die from irreparable damage to the PA or left bronchus during the slip-knot placement. Mice that did not survive the surgery or the reperfusion period due to technical complications in the surgical procedure (predominantly, left bronchus or left PA injury) were excluded from the study.

### Caspase 1 Inhibitor and IL-1β Blocking Antibody *In Vivo* Treatment

Wild-type C57B/L6 mice were pretreated with either a caspase 1 inhibitor (ac-YVAD-cmk peptide, Bachem Americas, Inc., Torrence, CA, USA) given approximately 30 min before left lung ischemia or an IL-1β blocking antibody (AB-401-NA; R&D Systems, Minneapolis, MN, USA) given 18–22 h before left lung ischemia. The YVAD peptide was prepared per the manufacturer’s instructions. Briefly 5 mg of powder was dissolved in DMSO to create a 50 mg/mL stock solution, which was then diluted in PBS to 50 µg/mL and 1 mg/kg was given to each mouse. Vehicle control was prepared similarly but without adding the YVAD peptide.

For the IL-1β blocking antibody, each mouse received 100 µg IP. Control mice received a control IgG polyclonal goat antibody (100 µg IP). Lungs and plasma were collected 1 h after reperfusion as described earlier.

### *Escherichia coli* Peritonitis in Wild-type following Lung IR

A murine model of *E. coli* peritonitis was introduced into C57BL/6 mice that received 30 min ischemia followed by 3 h reperfusion. The mice were then infected with *E. coli* intraperitoneally and 2 h later sacrificed and plasma and lungs collected for measurement of circulating levels and local production of cytokines and chemokines, respectively.

### *E. coli* Pneumonia in Wild-type and Inflammasome KO Mice

A murine model of *E. coli* pneumonia was generated as follows: *E. coli* O111 was recovered from frozen stocks by streaking on an LB agar plate. Following overnight growth, single colonies were used to inoculate 5 mL of LB broth and grown at 37 C overnight with shaking. The following day, 2.5 mL of the overnight culture was used to inoculate 50 mL of LB broth and grown for 4–6 h at 37°C with shaking. Once the optical density was between 0.7 and 1.0, the cultures were spun down at 3,000 rpm for 10 min, rinsed in sterile PBS, and then resuspended in LB broth containing glycerol (30%) and aliquots were made for infection experiments and flash frozen on dry ice, followed by storage at −80°C. Sample aliquots were thawed to confirm the stability of CFU counts before using them in animal experiments.

*Escherichia coli* was instilled in specific mice (wild type and various knockouts as described) *via* intratracheal (IT) instillation of 2 µL/g mouse weight (60 µL for a 30 g mouse) and the CFU corresponding to this was measured with each thawed aliquot (approximately 4–5 × 10^9–10^ given IT to each mouse). Mice were first anesthetized with 3% isoflurane in an anesthetic chamber with oxygen as the carrier gas. After loss of pedal reflex, the mouse was quickly placed on an intubating slide. The vocal cords were visualized using an external fiberoptic light source and a curved forceps to lift the tongue up and to the left. *E. coli* was then administered using a 27 g syringe with a curved catheter (0.6 mm diameter) on the needle. Successful instillation was accompanied by a characteristic change in the spontaneous breathing pattern of the mouse. For mice that received the IR surgery, the *E. coli* IT instillation was performed as described above at 3 h after the start of the reperfusion period.

Following *E. coli* IT instillation, the mice were allowed to recover from anesthesia and returned to their cages and to a BSL2 room. These mice were observed thereafter at 1 h later, and then daily until the 48 h endpoint was reached.

Plasma and organs were then collected as described above and the organs were homogenized (Tissue-Tearor-Biospec Products, Bartlesville, OK, USA) in sterile PBS. Serial 10-fold dilutions were then performed to measure CFU after overnight incubation of LB agar plates at 37°C.

### Quantitative Reverse Transcription Real-time Polymerase Chain Reaction

TaqMan-specific inventoried gene primers for glyceraldehyde 3-phosphate dehydrogenase (GAPDH), beta actin, interleukin (IL)-6, were used to measure the message levels of these genes in lung tissue (Life Technologies, Carlsbad, CA, USA).

Lung tissue was homogenized (Tissue-Tearor) and total RNA isolated using Trizol^®^. We used the High Capacity RNA-to-cDNA reverse transcription Kit using 1 µg messenger RNA per reaction (Life Technologies). Quantitative real-time polymerase chain reaction was performed using the ABI Prism 7000 Sequence Detection System (Life Technologies). Run method: polymerase chain reaction activation at 95°C for 20 s was followed by 40 cycles of 1 s at 95°C and 20 s at 60°C.

The average threshold count (Ct) value of 2–3 technical replicates was used in all calculations. The average Ct values of the internal controls (GAPDH, beta actin) were used to calculate ΔCt values for the array samples. Data analysis was performed using the 2^−ΔΔCt^ method, and the data were corrected for statistical analysis using log transformation, mean centering, and autoscaling (35–37).

### Sandwich ELISA

Concentrations of IL-6, IL-12p70, chemokine (C–C motif) ligand (CCL) 2/monocyte chemotactic protein (MCP) 1, chemokine (C–X–C motif) ligand (CXCL) 1 in mouse plasma were determined using the corresponding mouse Quantikine kits (R&D Systems, Minneapolis, MN, USA). All assays were performed according the manufacturer’s supplied protocol. Standard curves were generated and used to determine the concentrations of individual cytokines or chemokines in the sample.

### Dihydrorhodamine (DHR) Assay

Neutrophil superoxide production was assessed to determine whether neutrophils recruited following IR exhibited an activated phenotype. DHR 123 (Molecular probes) is a highly sensitive reactive oxygen species (ROS)-detection probe (38, 39) and thus, was chosen to assess intracellular superoxide as previously described (40) with modifications.

The left lower lung lobes were finely chopped with sterile scalpels and placed in HBSS + 0.1% BSA, passed through a 40 µm filter, and spun down. Collagenase digestion of the lung tissue and Fc receptor blocking was not done due to concerns that either process could potentially activate neutrophils. Red blood cells (RBCs) were lysed by incubating the cell suspension in RBC Lysis Buffer for 5 min at RT. Cells were again spun down, resuspended in HBSS + 0.1% BSA and counted. The cells were then incubated with APC-conjugated Ly6G antibody (clone 1A8, Biolegend) for 30 min at 4°C to stain neutrophils. Following this, cells were washed once with HBSS + 0.1% BSA and once with PBS-EGG (1 mM EDTA, 0.09% d-glucose, 0.05% gelatin in PBS). DHR 1, 2, 3 (10 µM; Molecular Probes) was added to the cells and incubated for 30 min at 37°C. The reaction was stopped by adding excess PBS-EGG at 4°C and incubating for 10 min. Cells were spun down, resuspended in PBS-EGG at 4°C and rhodamine fluorescence analyzed on a flow cytometer within 30 min.

### *In Vivo* Imaging (IVIS^®^)

B6 Albino mice were either subjected to IR surgery (as described above; 30 min ischemia, 3 h reperfusion) or IT LPS administration (1 mg/kg) and were imaged at the time points noted. Imaging was conducted on the IVIS^®^ Spectrum Instrument (PerkinElmer) as previously described (41). Briefly, prior to each imaging time point, mice were injected IV with 200 mg/kg luminol-R [for example, 125 mL of 40 mg/mL luminol (Sigma-Aldrich) mixed with 12.5 µL of 100 pmol QD800 quantum dots (Life Technologies)] and luminescence imaging was performed with an open filter for 5 min. 1 mg/kg LPS was injected intratracheally as well following left lung IR to activate dormant neutrophils.

### Lung Processing for Fluorescence Microscopy

tdTomato/MRP8 Cre mice that express the tdTomato protein under the neutrophil-specific MRP8 promoter were used to visualize the localization of neutrophils in the lung. Following IR (described above), fluorescein-labeled Lycopersicon esculentum lectin solubilized in phosphate-buffered saline (1 mg/ml), was injected as a 100 µl bolus into the tail vein prior to euthanasia to visualize the lung vasculature. Lungs were fixed by IT inflation with Z-fix (buffered zinc-formalin fixative, Anatech Ltd., Battle Creek, MI, USA) at 25 cm pressure, stored at 4°C overnight in Z-fix, washed in PBS, and then transferred to 30% sucrose in PBS prior to OCT embedding. Lungs were embedded in a mixture of 30% sucrose in PBS and OCT (1:1) and then frozen on dry ice. OCT-embedded sections (5 µm thickness) were stained with 4′,6-diamidino-2-phenylindole dihydrochloride (DAPI, Sigma-Aldrich, St. Louis, MO, USA) to visualize the nuclei.

### RNA Scope^®^*In Situ* Hybridization

Lungs from wild-type C57BL/6 or C3H mice that underwent lung IR (60 min ischemia, 1 h reperfusion) or LPS challenge (10 mg/kg IV for 1 h) were fixed in formalin and paraffin sections were stained with specific RNA probes for IL-1β and other cell markers, such as hopx (AT1 cells), surfactant C (AT2 cells), CD11c (AMs and dendritic cells). RNA *in situ* hybridization for IL-1β mRNA and other genes as described earlier was performed manually using the RNAscope^®^ HD Red Reagent Kit (Advanced Cell Diagnostics Pharma Assay Services, Newark, CA, USA) according to the manufacturer’s instructions. Briefly, 5 µm formalin fixed, paraffin-embedded tissue sections were pretreated with heat and protease prior to hybridization with the target oligo probes. Preamplifier, amplifier and AP-labeled oligos were then hybridized sequentially, followed by chromogenic precipitate development. Each sample was quality controlled for RNA integrity with a positive control probe specific to the housekeeping gene peptidylprolyl isomerase B and for background with a negative control probe specific to bacterial *Bacillus subtilis* gene dihydrodipicolinate reductase (dapB). Optimization of pretreatment conditions was performed to establish the maximum signal to noise ration. Specific RNA staining signal was identified as black, red, or blue-green punctate dots. Samples were counterstained with Gill’s Hematoxylin with nuclei appearing light purple (42, 43).

### Statistical Analysis

Data in the figures are expressed as mean ± SD. Data from *in vivo* studies comparing two conditions were analyzed using two-tailed nonparametric Mann–Whitney analyses. GraphPad Prism was used for statistical analyses (GraphPad Software, La Jolla, CA, USA). For all *in vivo* and *in vitro* experiments, *P* values <0.05 were considered significant. *P* values are represented as follows in the figures: *<0.05; **<0.01; ***<0.001; and ****<0.0001. Experiments were repeated two or more times, as indicated in the figure legends.

### Study Approval

All animal studies were ethically and methodologically approved by the Institutional Animal Care and Use Committee at the University of California, San Francisco, CA, USA.

## Results

### Lung IR Inflammation Is Regulated by NLRP3-Containing Inflammasomes *In Vivo*

**Figure 1:**
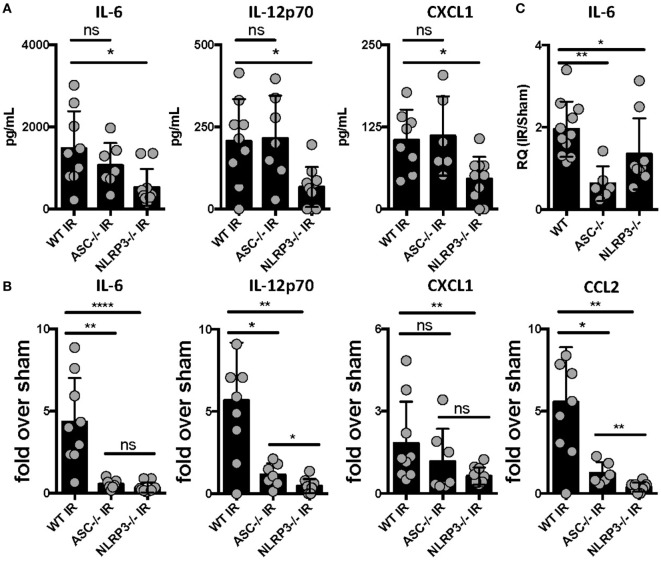
Lung ischemia–reperfusion (IR) inflammation depends on the presence of the NOD-, LRR-, and pyrin domain-containing 3 (NLRP3) inflammasome *in vivo*. Wild-type, ASC KO, and NLRP3 KO mice (all BL/6 background) were either subjected to sham or left lung IR surgery and following 1 h of reperfusion, plasma was collected and represented cytokines and chemokine levels measured by enzyme-linked immunosorbant assay (ELISA) and presented as **(A)** absolute values of cytokine levels or **(B)** fold induction over sham. **(C)** Left lungs from the ASC KO and NLRP3 KO mice depicted in **(A,B)** were homogenized, RNA isolated, and mRNA levels measured by qPCR [normalized to glyceraldehyde 3-phosphate dehydrogenase (GAPDH) and Actin] and presented as fold induction of interleukin (IL)-6 mRNA in IR over sham in relative quantity (RQ) units. Data in the figures are expressed as mean ± SD. Data from *in vivo* studies comparing two conditions were analyzed using two-tailed nonparametric Mann–Whitney analyses.

The mechanisms by which sterile injury results in inflammation and how these differ from mechanisms underlying the host inflammatory response to infection are very active topics of research currently. Having hypothesized that the NLRP3 inflammasome would be required for the generation of sterile inflammation following lung IR, we subjected ASC KO and NLRP3 KO mice that lack either all inflammasome subtypes or just NLRP3 inflammasomes, respectively, to 1 h of ischemia followed by 1 h of reperfusion. We then quantified plasma and tissue levels of inflammatory cytokines. In these and following experiments, and as verified in prior publications (25, 34), we measured IL-6 and chemokine levels as surrogate markers of sterile lung IR inflammatory response that includes subsequent neutrophil recruitment and lung edema. We found that while NLRP3 KO mice following lung IR expressed cytokine levels that were significantly lower compared to wild-type mice, ASC KO mice did not (Figure 1A). To account for reported and observed elevations in baseline levels of inflammatory markers in ASC KO mice (44–46), we normalized these cytokine levels to those from mice that underwent a sham procedure. By doing so, we observed a significantly weaker induction of inflammatory cytokines and chemokines in both ASC KO and NLRP3 KO mice (Figure 1B). We confirmed this at the mRNA level in lung tissue as well (Figure 1C). Together these data strongly support the conclusion that sterile lung injury initiates downstream inflammatory cytokine release through the activity of the NLRP3 inflammasome.

### Caspase 1 Presence and Activation Is Required for Full IR-Induced Inflammation *In Vivo*

**Figure 2:**
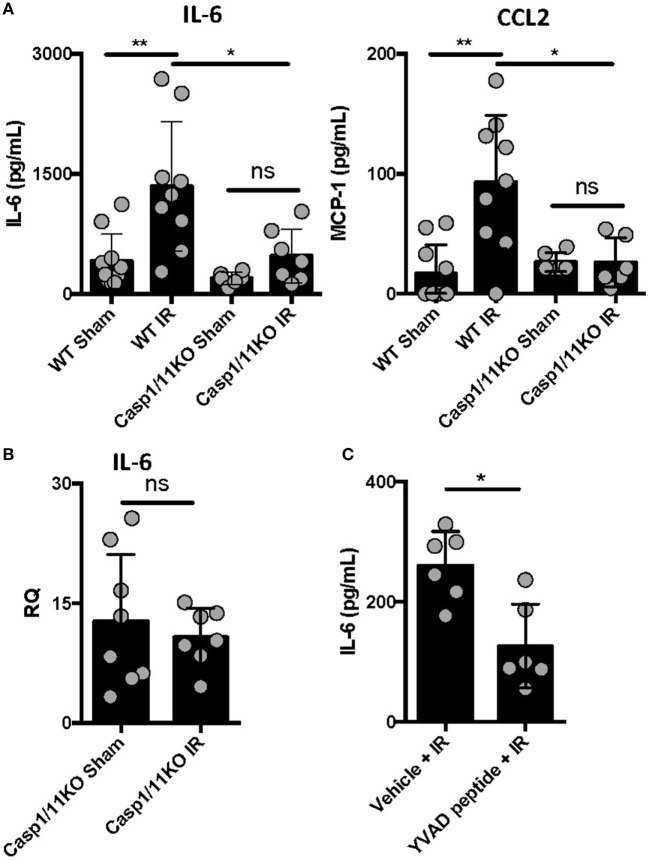
Caspase 1 presence and activation is required for full ischemia–reperfusion (IR)-induced inflammation *in vivo*. **(A)** Wild-type (BL/6 background) and Caspase 1/11 KO (129 background) mice were subjected to sham or left lung IR surgery and interleukin (IL)-6 and CCL2 (MCP-1) levels measured by enzyme-linked immunosorbant assay (ELISA) 1 h after reperfusion. **(B)** Left lungs from the Caspase 1/11 KO mice depicted in Figure 5A were homogenized, RNA isolated, and mRNA levels measured by qPCR [normalized to GAPDH and Actin]. **(C)** Wild-type mice (BL/6 background) were pretreated with YVAD peptide (Caspase 1 inhibitor) and then 30 min later subjected to IR; at 1 h reperfusion, plasma was collected and IL-6 levels measured by ELISA. Data in the figures are expressed as mean ± SD. Data from *in vivo* studies comparing two conditions were analyzed using two-tailed nonparametric Mann–Whitney analyses.

We previously reported that IL-1β is produced following ventilated lung IR and that *in vitro* macrophage derived IL-1β can augment endothelial cell IL-6 production (25). To further confirm the importance of IL-1β in lung IR inflammation, we used genetic and pharmacologic tools that target inflammatory caspases so as to disrupt the processing of IL-1β into its active form. Compared to wild-type mice, caspase 1/11 KO mice following IR displayed a significantly muted increase in levels of secreted IL-6, CCL2 (MCP-1), and IL-1β compared to sham (Figure 2A and data not shown) and this was confirmed at the tissue mRNA level (Figure 2B). Furthermore, the caspase 1-specific inhibitor peptide, YVAD, was able to significantly reduce IL-6 production following IR compared to a vehicle control (Figure 2C). Overall, we conclude that inflammatory caspases are required for lung IR inflammation.

### IL-1β Activity Regulates Lung IR Inflammation and IL-1β Is Located in Association with AT2 Epithelial Cells *In Vivo*

**Figure 3:**
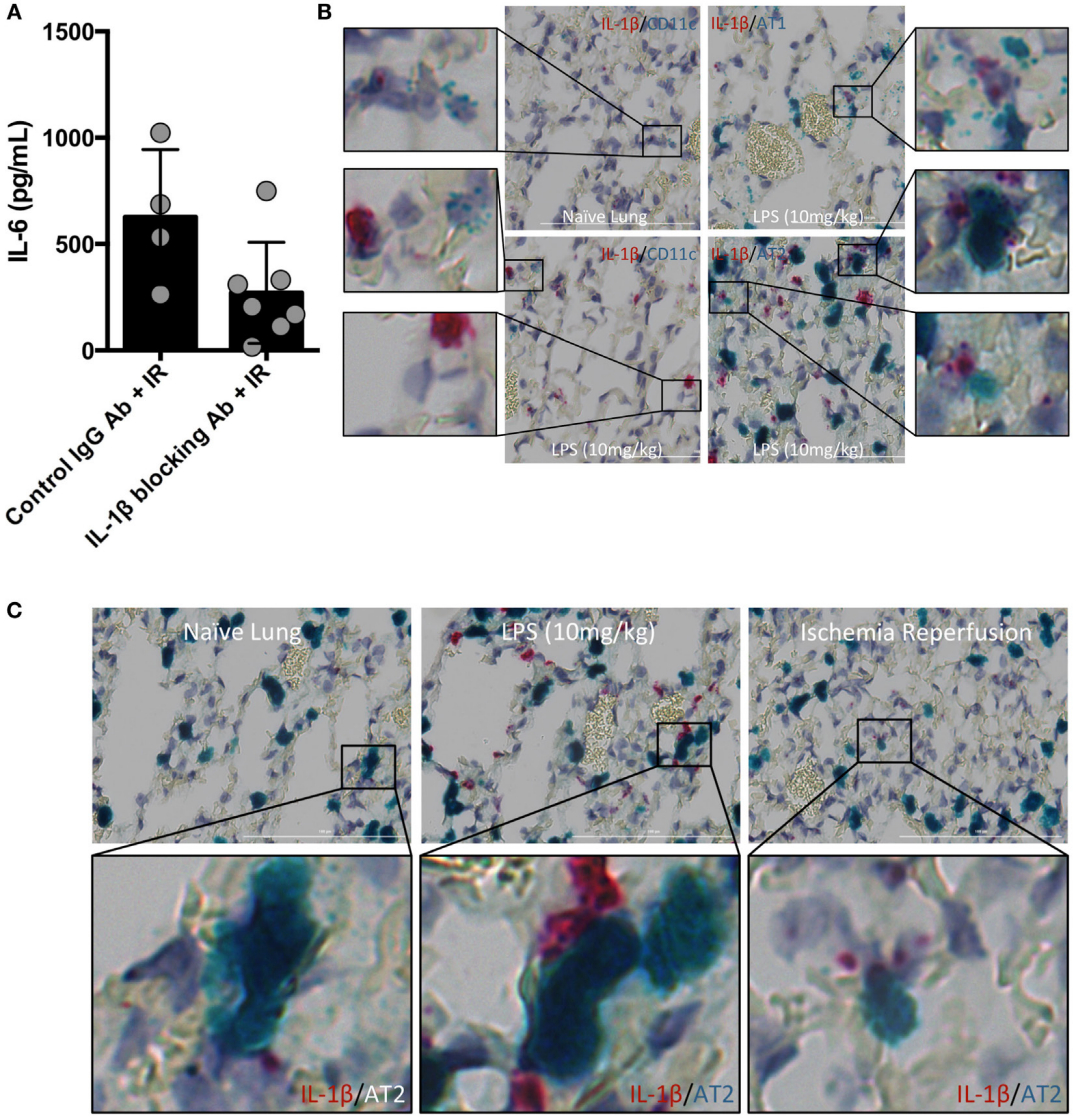
Interleukin (IL)-1β activity drives lung ischemia–reperfusion (IR) inflammation and originates from alveolar type 2 (AT2) epithelial cells. **(A)** Wild-type mice (BL/6 background) were pretreated with IL-1β blocking antibody and then 18 h later subjected to IR; at 1 h reperfusion, plasma was collected and IL-6 levels measured by enzyme-linked immunosorbant assay (ELISA). Data in the figures are expressed as mean ± SD. Data from *in vivo* studies comparing two conditions were analyzed using two-tailed nonparametric Mann–Whitney analyses. **(B)** Wild-type mice (naive, treated with LPS 10 mg/kg IV) were sacrificed 1 h after surgery/treatment and lungs fixed and stained with RNA probe pools (RNAScope™) as indicated in the figure. **(C)** Focusing only on the AT2 cells and IL-1β under naive, LPS treated and IR conditions, the IL-1β RNA signal was closely associated with that for Surfactant C (produced by AT2 cells).

Inflammasome and caspase activity can result not only in IL-1β production but also IL-18, and perhaps even IL-33. We next focused on testing whether IL-1β activity was important *in vivo* in lung IR inflammation. To do so, we used an IL-1β blocking antibody and observed a reduction in the levels of IL-6 produced following IR compared to an IgG control (Figure 3A). These data suggest that IL-1β is a key early signal generated after lung IR.

The identity of the cell type(s) that produce(s) IL-1β in the lung *in vivo* has not been definitively established. Data deposited in the Immgen and BioGPS/Gene Atlas databases report that many different cell types can produce IL-1β including bronchial epithelial cells, smooth muscle, dendritic cell (DC) subsets, monocytes and macrophages, neutrophil subsets, AMs, and alveolar epithelium. While it is clear that many lung cell types can produce IL-1β *in vitro*, and the airway epithelium has been implicated in a few reports, few if any studies have relied on examining lung cells in their native *in vivo* tissue context for IL-1β expression. Since the inflammasome is a highly regulated signaling complex and the release of IL-1β requires strict temporal and spatial control, we hypothesized that IL-1β release would be restricted to one or a few specific cell types. Candidates in the lung included AM and the cells that form the alveolar epithelium (type 1 and type 2 alveolar epithelial cells, namely, AT1 and AT2 cells), other lung macrophages or DCs. To identify the type of lung cell(s) in which the inflammasome activation, caspase and IL-1β processing takes place, we used an *in situ* hybridization methodology with pools of specific IL-1β, and other RNA probes. The IL-1β containing cell in resting lungs appeared to line the alveolar space (Figure S1 in Supplementary Material). We believe that these IL-1β-expressing cells participate in the sensing of injury generated by lung IR and trigger the cascade of events leading to mature IL-1β release, IL-6 production, neutrophil recruitment, and overall lung inflammation. Even following *in vivo* LPS challenge (employed as a means to further induce IL-1β mRNA expression), the increased lung IL-1β expression appeared to still localize within a similar cell type lining the alveolar space (Figure S2 in Supplementary Material). IL-1β mRNA signal was detected at low levels in isolated naive mouse lung, and this signal was increased following IL-1β gene induction after both LPS challenge and lung IR, even if the LPS response was far more robust (Figures 3B,C). Using specific probe pools for genes specific to alveolar epithelial cells (Hopx for AT1 and Surfactant C for AT2), macrophages (F4/80), AMs (CD11c), DCs (SiglecH, CD103) revealed that the IL-1β signal colocalized most closely with surfactant C made by AT2 epithelial cells (Figure 3B and data not shown). Of note, we did also observe some weak LPS-induced IL-1β expression in AMs.

### Downstream IL-1 Signaling Is Also Required for Full IR-Induced Inflammation *In Vivo*

**Figure 4:**
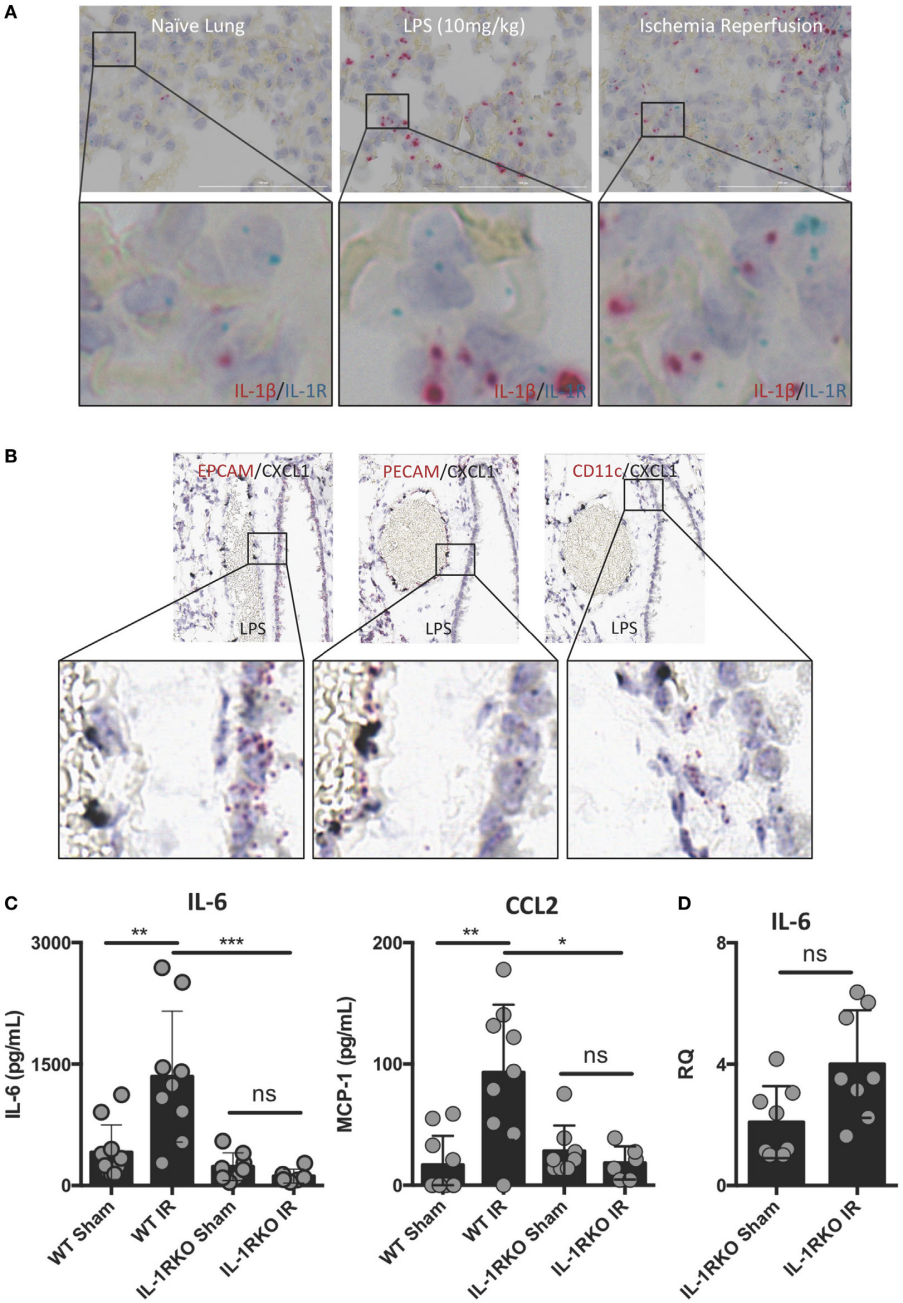
Interleukin (IL)-1β activity likely acts locally within lung and IL-1 signaling is required for full ischemia–reperfusion (IR)-induced inflammation *in vivo*. **(A)** IL-1R expressing cells exist within the lung in close conjunction with IL-1β producing cells in the naive, LPS-stimulated, and post-IR lung tissue. **(B)** chemokine (C–X–C motif) ligand (CXCL) 1 is expressed solely in the lung endothelium (PECAM+) and not the epithelium (EPCAM+) or alveolar macrophages (CD11c+) in WT mice 1 h following LPS (10 mg/kg IV) challenge. **(C)** Wild-type and IL-1R KO mice were subjected to sham or left lung IR surgery and IL-6 and monocyte chemotactic protein (MCP)-1 levels measured by enzyme-linked immunosorbant assay (ELISA) 1 h after reperfusion. **(D)** Left lungs from these same IL-1R KO mice were homogenized, RNA isolated, and mRNA levels measured by qPCR [normalized to GAPDH and Actin]. Data in the figures are expressed as mean ± SD. Data from *in vivo* studies comparing two conditions were analyzed using two-tailed nonparametric Mann–Whitney analyses.

Interleukin-1β often functions as a locally acting cytokine within the tissue where it was released. Consistent with this, the levels of IL-1β released systemically after lung IR are relatively low to undetectable [data not shown and Ref. (25)]. To examine whether downstream IL-1β signaling within the lung is important for lung IR inflammation, we investigated the expression of IL-1R in the lung and how IL-1R KO mice responded to lung IR. We observed dispersed IL-1R expression in multiple cell types in lung tissue, however, we consistently observed IL-1R expressing cells in close proximity to the IL-1β producing cell (Figure 4A). This supported the idea that IL-1β primarily acts as a locally signaling cytokine. In contrast, the neutrophil attracting chemokine, CXCL1, was induced in lung endothelial cells (Figure 4B) and easily detected systemically (25). As shown in Figure 4C, unlike wild-type mice, IL-1R KO mice were profoundly unable to induce IL-6 or CCL2 (MCP-1) production following IR. This was further confirmed at the tissue mRNA level (Figure 4D). These findings are consistent with a model placing IL-1β production and IL-1R engagement upstream of IL-6 and chemokine production. Furthermore, this conclusion is supported by the observation that IL-1R KO mice did not display significant neutrophil recruitment following lung IR compared to wild-type mice (data not shown). These data begin to shed light on the detailed choreography of intercellular signaling that occurs within the lung following injury.

### Lung IR Inflammation Recruits Dormant Neutrophils

**Figure 5:**
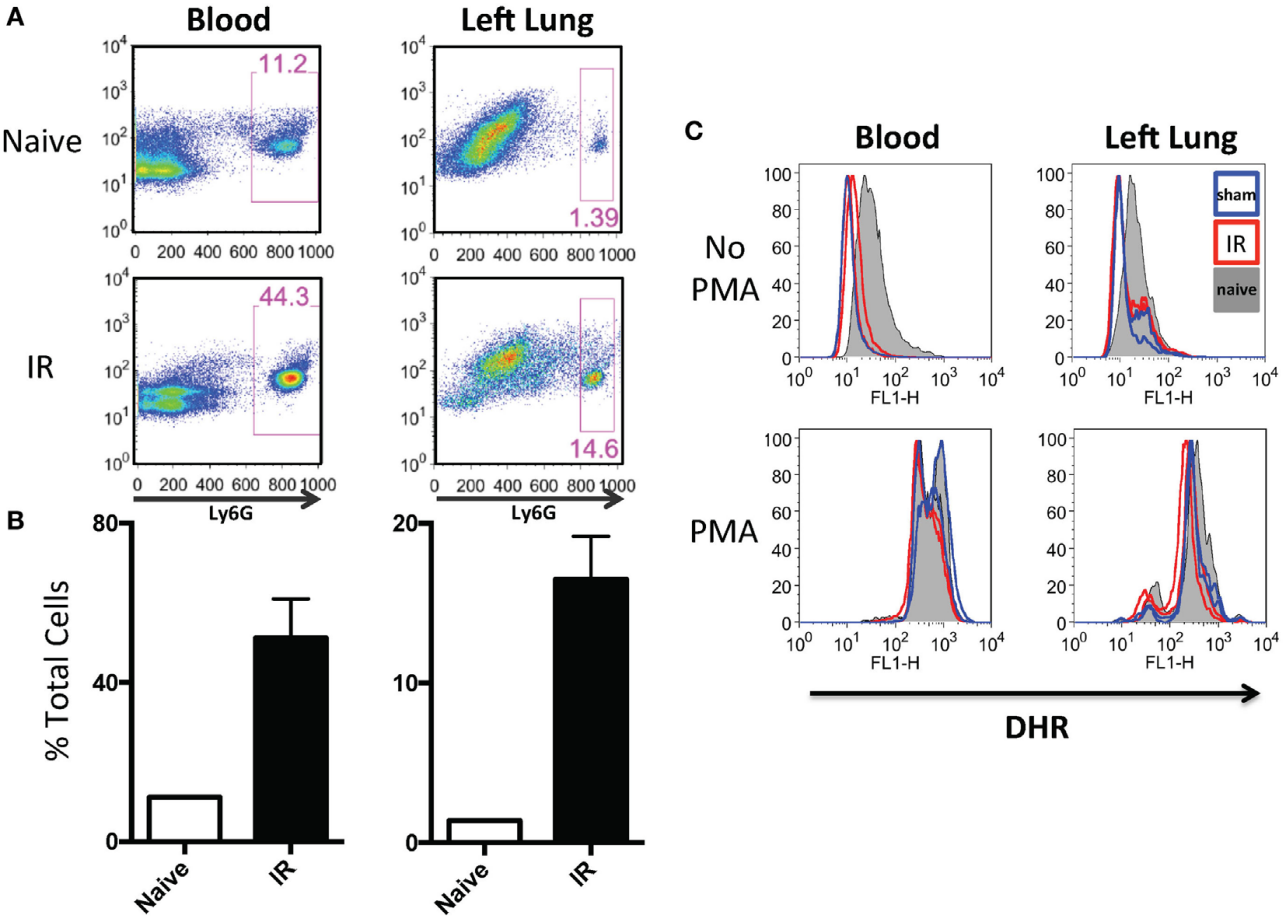
Lung ischemia–reperfusion (IR) recruits dormant neutrophils. **(A)** Peripheral blood and left lungs were collected from naive mice or mice that underwent left lung IR. Lung single cell suspensions were prepared for flow cytometry. Neutrophils frequencies (Ly6G+) in the circulation and left lung are shown. **(B)** Quantification of the neutrophil expansion/recruitment in left lung and blood. **(C)** Blood and lung cell suspensions were treated with dihydrorhodamine (DHR) and reactive oxygen species (ROS) production was measured in Ly6G+ neutrophils (top). These same neutrophils were treated with phorbol ester (PMA) to activate the neutrophils [positive reactive oxygen species (ROS) control, bottom].

In our previously published work, we have shown that lung IR inflammation in WT mice manifested itself as rapid but transient increases in inflammatory cytokines, such as IL-6, MCP-1, etc. (peaking at 0–1 h postreperfusion) as well as transient neutrophilia (peaking at 3–6 h postreperfusion) (34). Given the uninjured appearance of the mouse lung 24 h following IR (34) and the lack of functional deficits in oxygen uptake as measured by pulse oximetry (unpublished data), we hypothesized that the early recruited neutrophils may not have degranulated or become fully activated. To tackle this, we first subjected mice to either LPS challenge, a sham or IR procedure and using flow cytometry, we determined that there was a 4–10-fold increase in neutrophil numbers (Ly6G+ cells) in the circulating blood and within the IR-affected lung (Figure 5A and quantified in Figure 5B) consistent with our previously published data (25). Of note, the sham mouse cohort also experienced some neutrophil recruitment to the lung, presumably in response to the incidental sterile injury generated by the surgical incision and lung manipulation during the sham procedure. In peripheral blood and single cell suspensions from the left lung, neutrophils were assessed for ROS production using an *in vitro* DHR assay. IR-recruited neutrophils, like sham surgery-recruited neutrophils had lower ROS generation even compared to naive mice that did not receive any surgery (Figure 5C, top two panels). However, when these neutrophils were challenged *in vitro* with phorbol ester (PMA), all subsets of neutrophils were able to strongly generate ROS (Figure 5C, bottom two panels).

**Figure 6:**
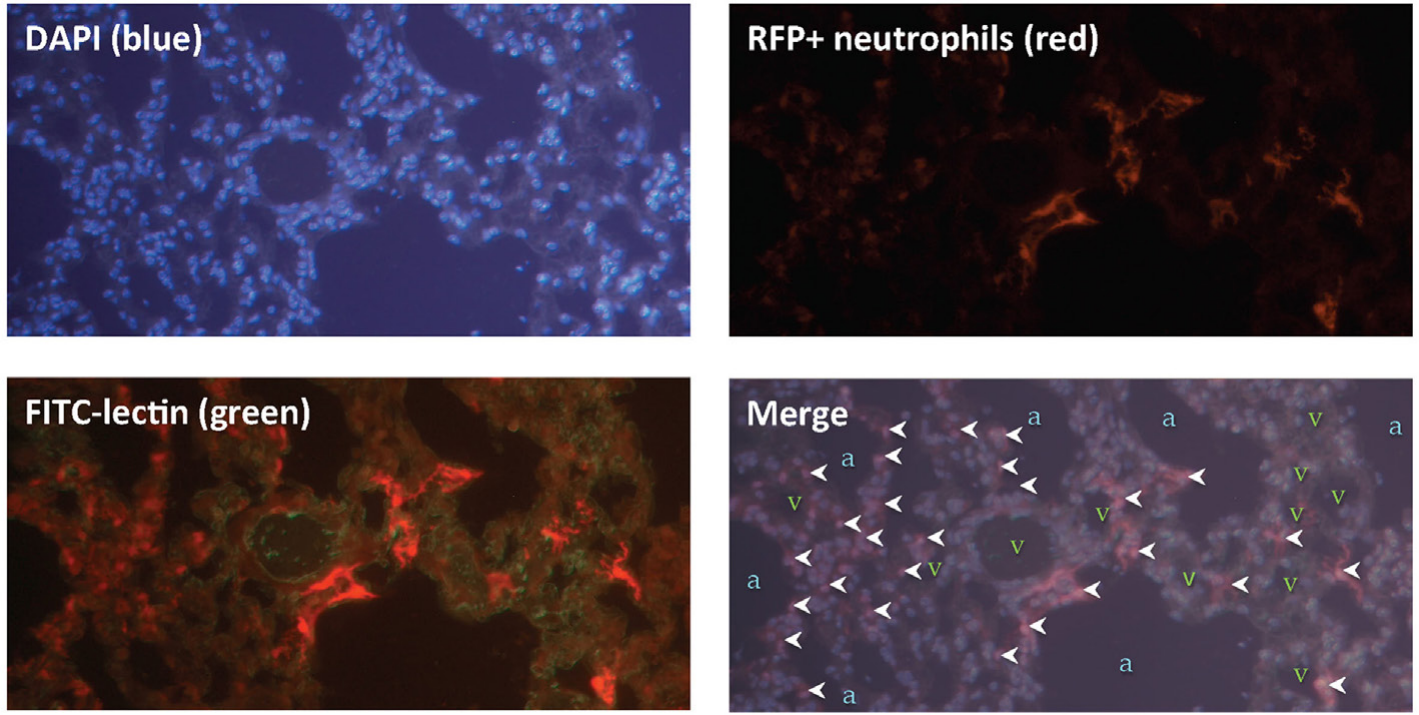
Lung ischemia–reperfusion (IR)-recruited neutrophils are located within lung parenchyma. Mice with tdTomato expressing neutrophils (under the control of the MRP8 neutrophil-specific promoter) were subjected to left lung IR. Three hours following reperfusion, FITC-lectin was injected IV and lungs were collected, fixed, and stained with DAPI. Upper panels show DAPI staining showing blue nuclei (upper left), and the RFP channel showing RFP+ neutrophils (upper right). Lower left panel shows the RFP channel and the FITC channel (outlining blood vessels in green). The lower right merge panel displays neutrophils (marked by white arrowheads), alveoli/air spaces (marked by “a”), and vasculature (marked by “v”).

Next, we sought to locate these neutrophils within the lung tissue following IR. Using mice that expressed red fluorescent neutrophils, we observed that the neutrophils were located within the lung parenchyma and not within the air spaces (Figure 6).

**Figure 7:**
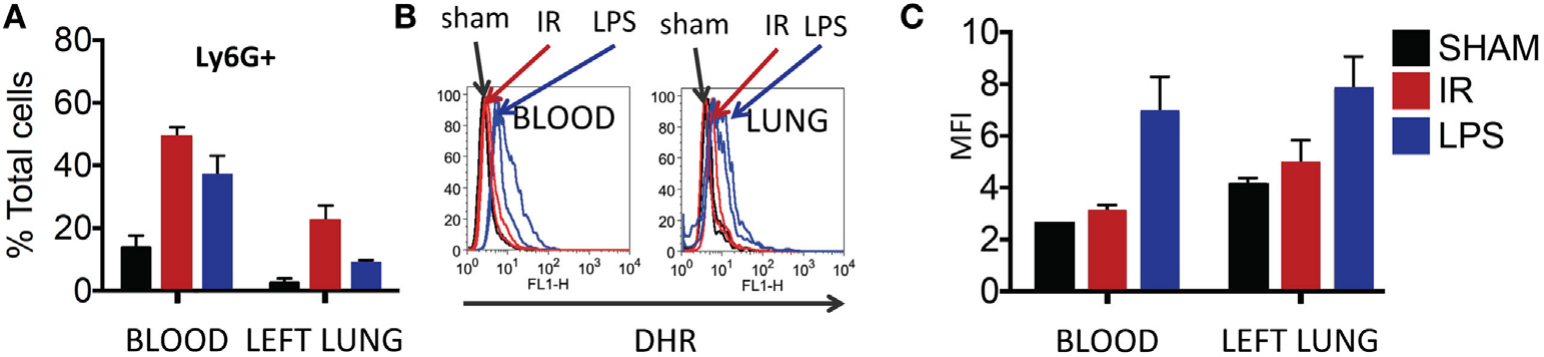
Dormant neutrophils recruited in lung ischemia–reperfusion (IR) are different to those recruited by LPS. The frequency and reactive oxygen species (ROS) activity of Ly6G+ neutrophils were assessed in peripheral blood and manually digested lung cell suspensions using flow cytometry. **(A)** An increase in neutrophil frequency in peripheral blood and lung was seen following both lung IR and LPS challenge. Neutrophils were selected from plasma or digested lungs as Ly6G+ cell population. **(B)** Neutrophils in the blood and lung of mice undergoing sham or IR surgery produce less ROS compared to neutrophils from LPS challenge. **(C)** Quantification of ROS production in neutrophils following LPS challenge versus sham or IR surgery.

Thereafter, we compared neutrophils recruited to the IR lung to those recruited by LPS. LPS challenge resulted in a comparable recruitment of neutrophils in blood and lung (Figure 7A), but these neutrophils produced significantly more ROS than those recruited in IR or sham surgeries (Figure 7B, and quantified in Figure 7C).

**Figure 8:**
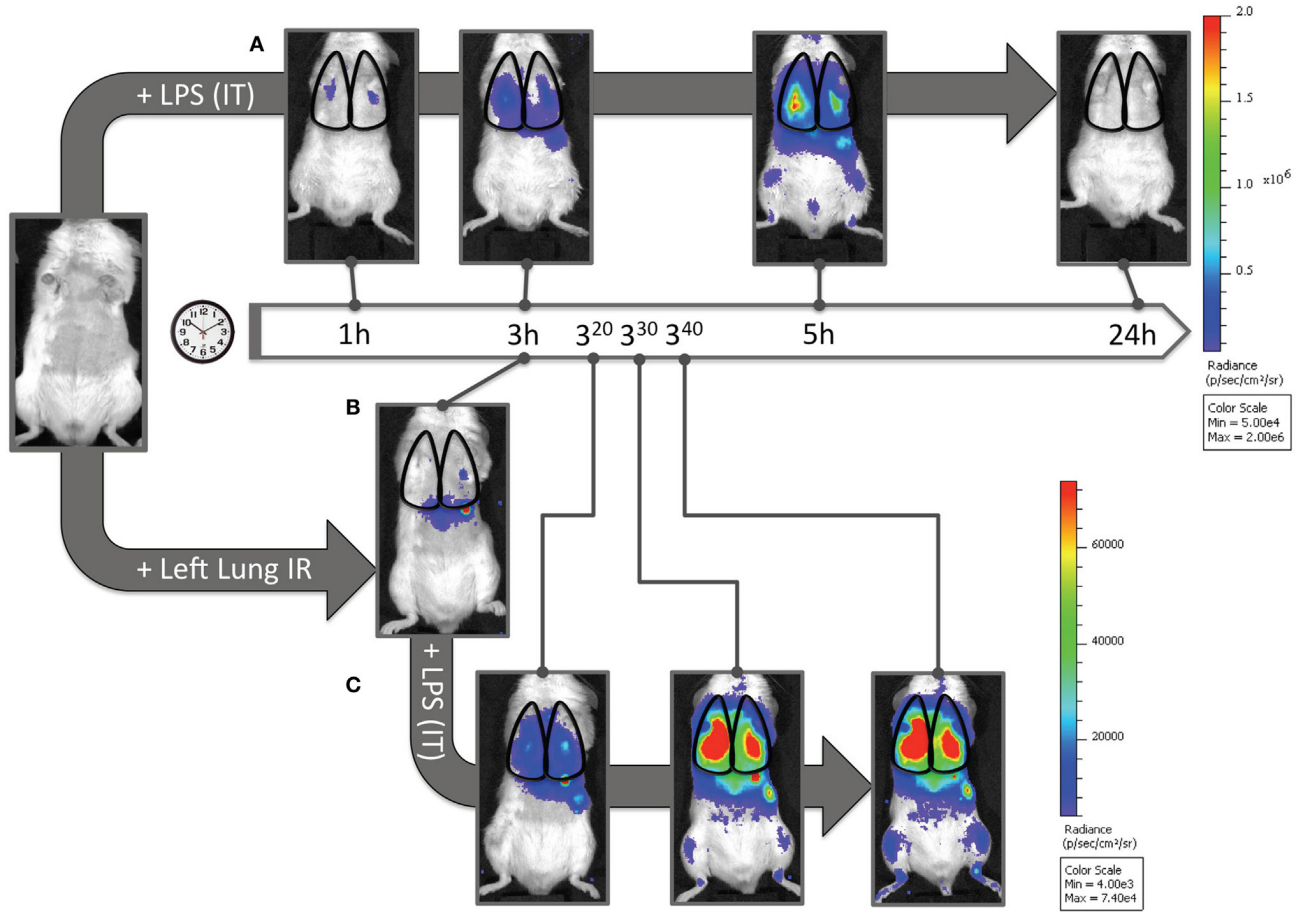
Dormant neutrophils recruited to lungs after ischemia–reperfusion (IR) can be activated *in vivo*. IVIS™ *in vivo* imaging was used to detect *in vivo* neutrophil activation using a luminol/Qdot probe that emits light in the presence of activated neutrophil MPO. **(A)** Albino C57BL/6 mice were challenged with intratracheal LPS (1 mg/kg), and at denoted time points (1, 3, 5, and 24 h) were imaged and activated MPO in recruited neutrophils was observed in the lung fields. The scale is provided to the right of the IVIS™ images. **(B)** Three hours after lung IR, negligible lung signal corresponding to activate neutrophil MPO was observed in mice. **(C)** However, after intratracheal LPS challenge (1 mg/kg), these mice rapidly expressed activated MPO 20–40 min after LPS administration [versus at 3–5 h in panel **(A)**]. The scale for **(B,C)** is provided to the right of the IVIS™ images. *Note 1*: activated MPO in macrophages is not detected using this luminol-Qdot probe; this methodology specifically detects neutrophil activated and released MPO (41). *Note 2*: an activated neutrophil signal in the stomach of mice after luminol-Qdot IV injection is commonly seen in this methodology.

Finally, we asked whether this recruitment of apparently “dormant” neutrophils occurred *in vivo* to rule out the possibility that our findings were an artifact of lung preparation or neutrophil activity alterations once the neutrophils were separated from the *in vivo* context of the lung and the host. To answer this question, we measured myeloperoxidase (MPO) activation in injured lung tissue *in vivo* using *in vivo* imaging (IVIS™). We had previously shown that the administration of luminol with quantum dots permitted the specific tracking of activated neutrophils (and not activated macrophages) to the lung in live mice following LPS administration (41). As seen in Figure 8A, when mice received LPS, a robust accumulation of activated neutrophils was detected in the lungs at 3 h and not at 1 h. In contrast, 3 h after reperfusion, IR surgery mice displayed no signal correlating to activated neutrophils in the lungs. Thus there appeared not be any activated MPO-producing neutrophils in the lungs of injured mice 3 h following IR (Figure 8B). However, to confirm the presence of these dormant neutrophils, we introduced LPS into the lung, as a tool to expose the presence of these cells. By virtue of activated MPO signal emerging in these IR mice rapidly within 20–40 min (Figure 8C), we conclude that while *in vivo* IR-recruited neutrophils in the lung are dormant, they are still capable of rapid activation/degranulation *in vivo* in the presence of pathogen-derived toxins like LPS.

### Lung IR Generated Inflammation Promotes Immune Defense against Bacterial Pneumonia

**Figure 9:**
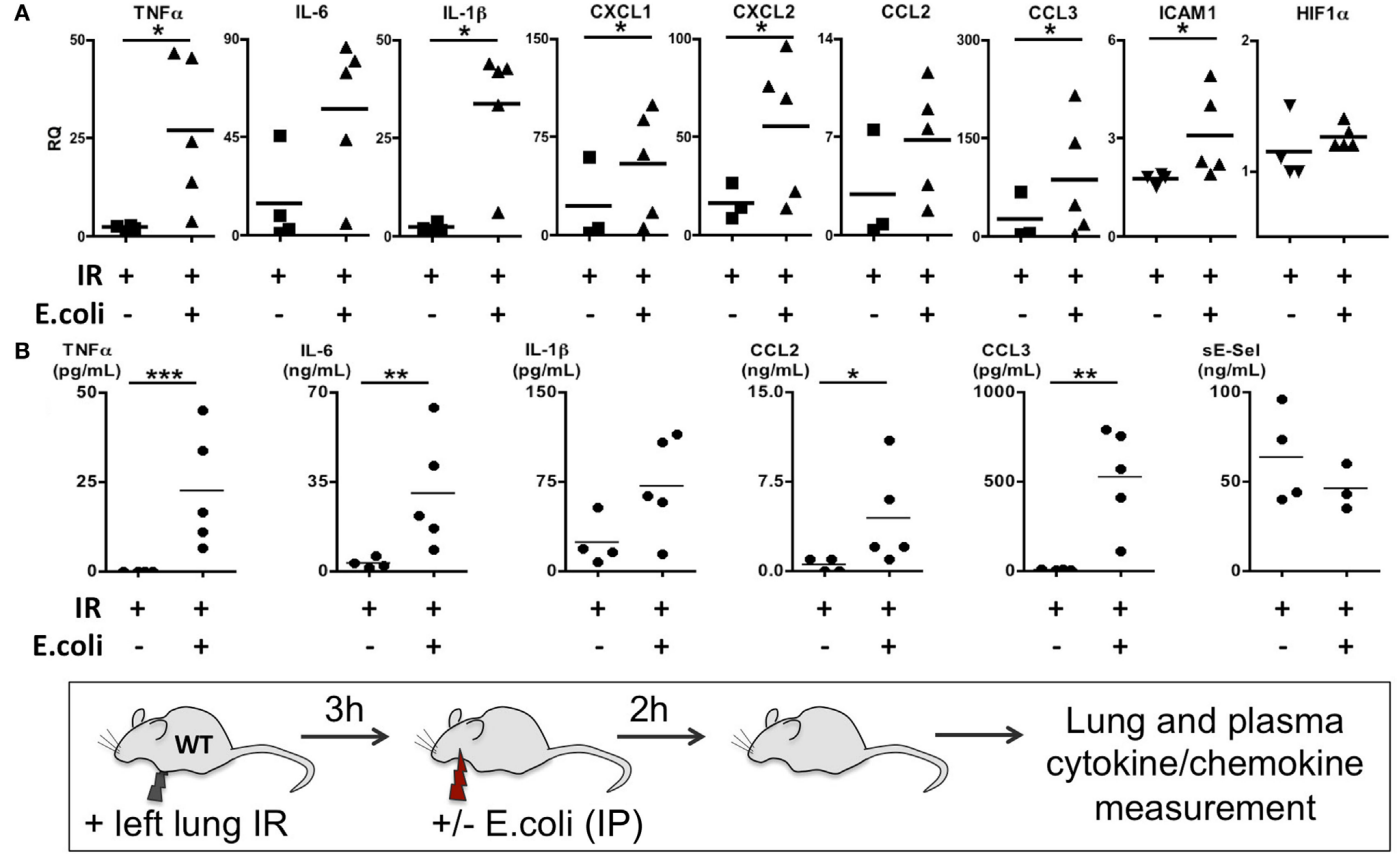
Coinfection following lung ischemia–reperfusion (IR) results in much higher local and systemic cytokine and chemokine production *in vivo*. Wild-type BL/6 mice received lung IR and following 3 h of reperfusion were either infected with IP instillation of *Escherichia coli* (5 × 10^7^ in 200 µL PBS) or vehicle. Two hours later, left lungs **(A)** and plasma **(B)** and were collected and cytokine/chemokine/adhesion molecules levels measured by qPCR **(A)** or enzyme-linked immunosorbant assay (ELISA) **(B)**. Data from *in vivo* studies comparing two conditions were analyzed using two-tailed nonparametric Mann–Whitney analyses.

**Figure 10:**
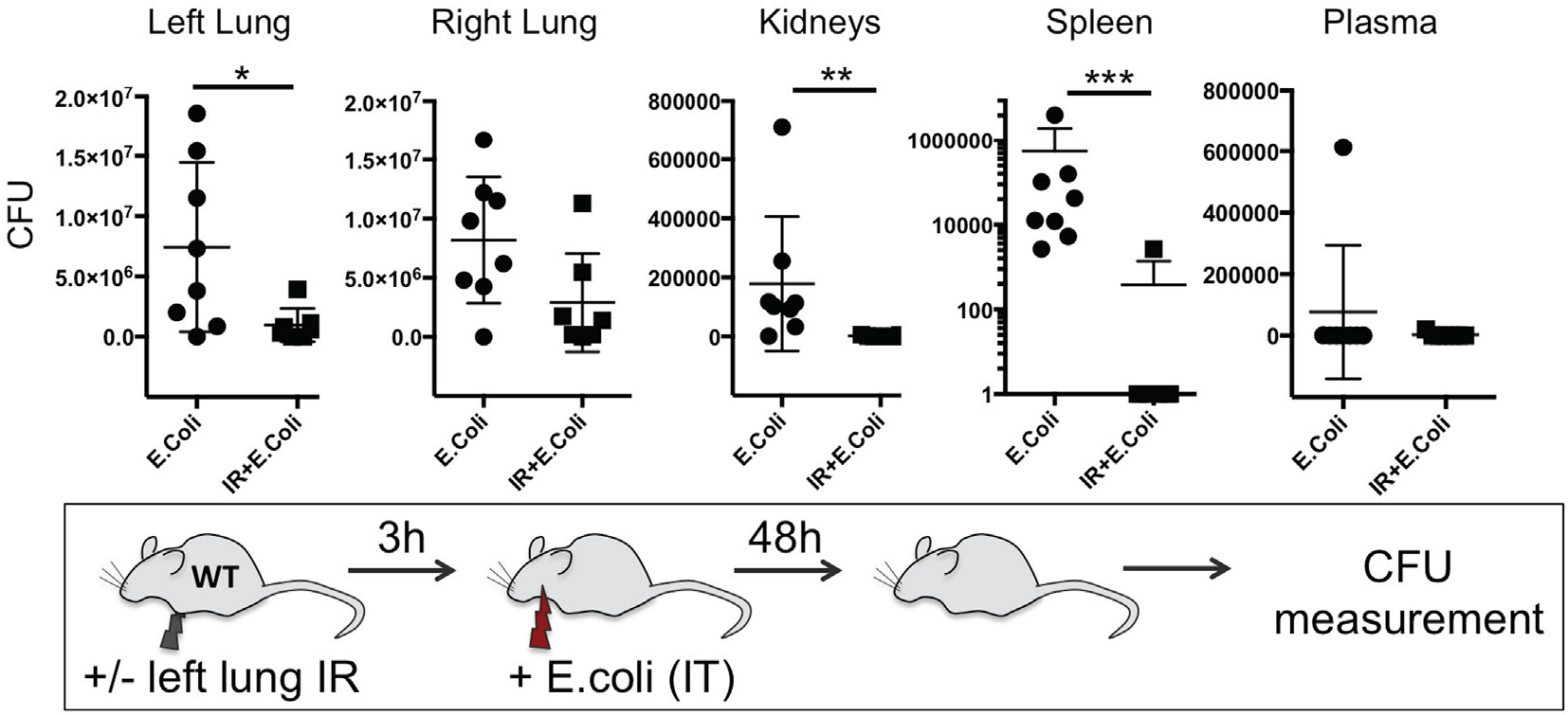
Lung ischemia–reperfusion (IR)-generated inflammation promotes clearance of bacterial pneumonia locally and reduces dissemination of infection in distal organs *in vivo*. Wild-type BL/6 mice either received lung IR or not followed 3 h later with intratracheal (IT) instillation of *Escherichia coli*. Approximately 48 h later, plasma and selected organs were collected, homogenized, and colony-forming unit (CFU) measured. For clarity, the Spleen data is shown as log transformed CFU with no detectable CFU set at 0.1 CFU. Each data point represents the CFU obtained from the respective sample from a single mouse. Data in the figures are expressed as mean ± SD. Data from *in vivo* studies comparing two conditions were analyzed using two-tailed nonparametric Mann–Whitney analyses.

Thus far we have shown that NLRP3 and IL-1β are key immune molecules activated following lung IR, and which result in the trafficking of minimally activated dormant neutrophils to the lung. Next, we attempted to define the role that these neutrophils and the creation of inflammatory conditions within the lung parenchyma might play in lung pathophysiology. It is highly likely that an evolutionary reason exists for why neutrophils are recruited in lung following IR but remain dormant. We speculated that host lung immunity evolved to anticipate pathogen presence during and/or after lung trauma, and may have done so by orchestrating the trafficking of neutrophils to the site of injury in the expectation of encountering pathogen. To test this hypothesis, we first subjected mice to lung IR and then exposed their lungs to *E. coli* to determine whether the prior presence of lung IR would result in early and amplified inflammatory signals generated in the lung. Introducing *E. coli* led to vastly greater cytokine and chemokine levels both within the lung and systemically in plasma compared to IR alone (Figure 9) and appeared to reduce the bacterial burden early (2 h) after infection (data not shown). To confirm whether this amplified inflammatory response was indeed beneficial, we tracked the course of infected mice subjected to preceding lung IR *vis-à-vi*s bacterial burden. Remarkably, we found a near total absence of bacteria locally at the site of IR and infection (left lung) as well as significant decreases in organs such as the kidney and spleen 48 h after infection (Figure 10). Notably, the plasma of both groups of mice was free from detectable bacteria even though there was evident large-scale bacterial seeding of multiple organ systems. These data indicate that naturally occurring IR inflammation may have a beneficial role to play in situations that involve the convergence of sterile injuries and infection, such as is often encountered in trauma.

### Enhanced Clearance of Bacterial Infection in the Presence of IR Inflammation Is NLRP3 Inflammasome Dependent

**Figure 11:**
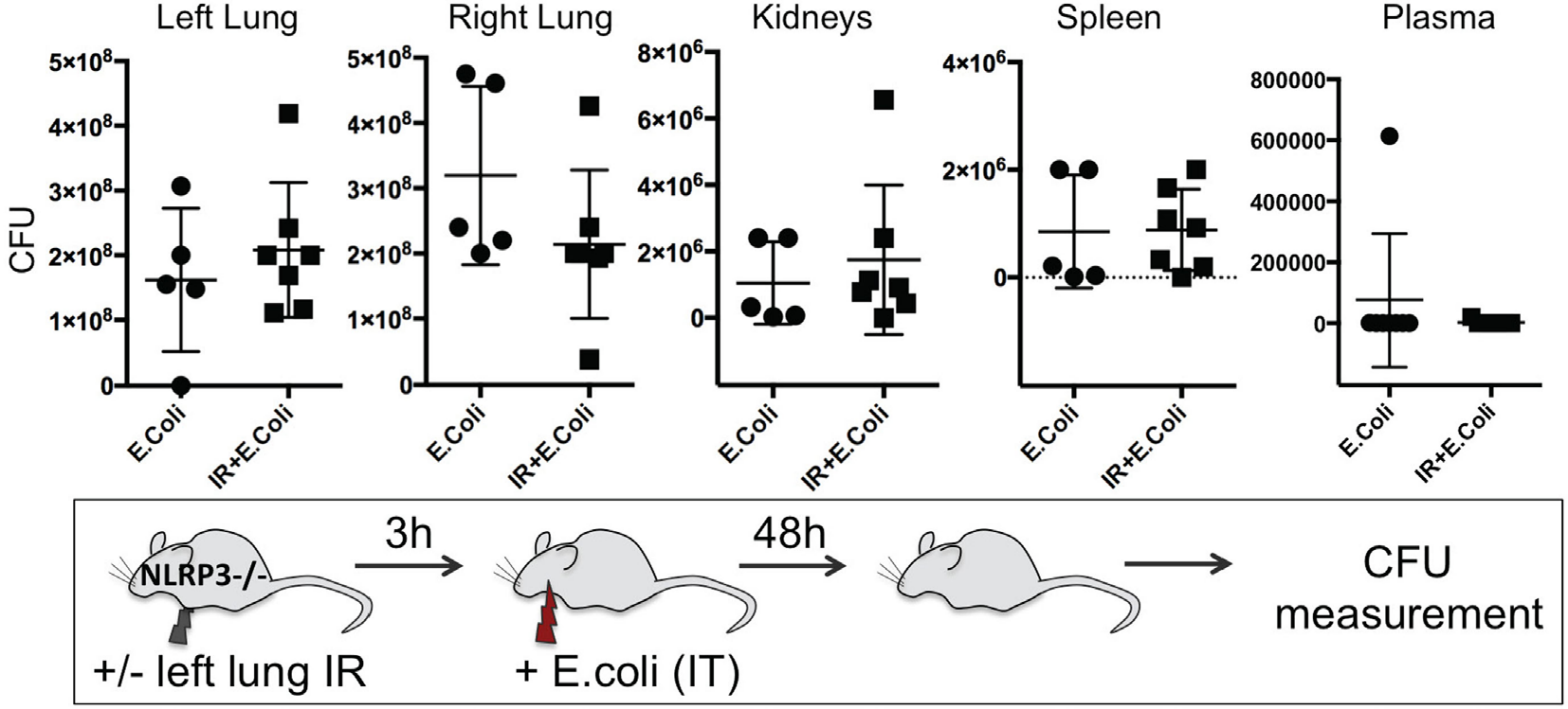
Enhanced clearance of bacterial infection *in vivo* is inflammasome dependent. NOD-, LRR-, and pyrin domain-containing 3 (NLRP3) KO mice either received lung ischemia–reperfusion (IR) or not followed 3 h later with intratracheal (IT) instillation of *Escherichia coli*. Approximately 48 h later, plasma and selected organs were collected, homogenized and colony-forming unit (CFU) measured. Each data point represents the CFU obtained from the respective sample from a single mouse. Data in the figures are expressed as mean ± SD. Data from *in vivo* studies comparing two conditions were analyzed using two-tailed nonparametric Mann–Whitney analyses.

To confirm the importance of the inflammasome in generating this protective lung IR inflammation against infection, we examined the ability of ASC KO and NLPR3 KO mice to contain an experimental bacterial *E. coli* pneumonia after lung IR. We found that mice deficient in all inflammasomes (ASC KO, Figure S3 in Supplementary Material), or specifically NLRP3-containing inflammasomes (NLPR3 KO, Figure 11) after IR, were unable to mitigate the course of the experimental *E. coli* pneumonia. As we had demonstrated earlier, these mice generated attenuated inflammatory responses following lung IR. Additionally, in some cases, preceding IR in these KO mice resulted in higher bacterial counts compared to the infection only group. Interestingly again, the plasma in these mice was also free of detectable pathogen suggesting that bacterial colonization of remote organs may not necessarily correlate with systemic blood-borne bacteremia.

### Beneficial Effects of Lung IR on Subsequent Control of Bacterial Pneumonia Can Persist for 24 h following Reperfusion

**Figure 12:**
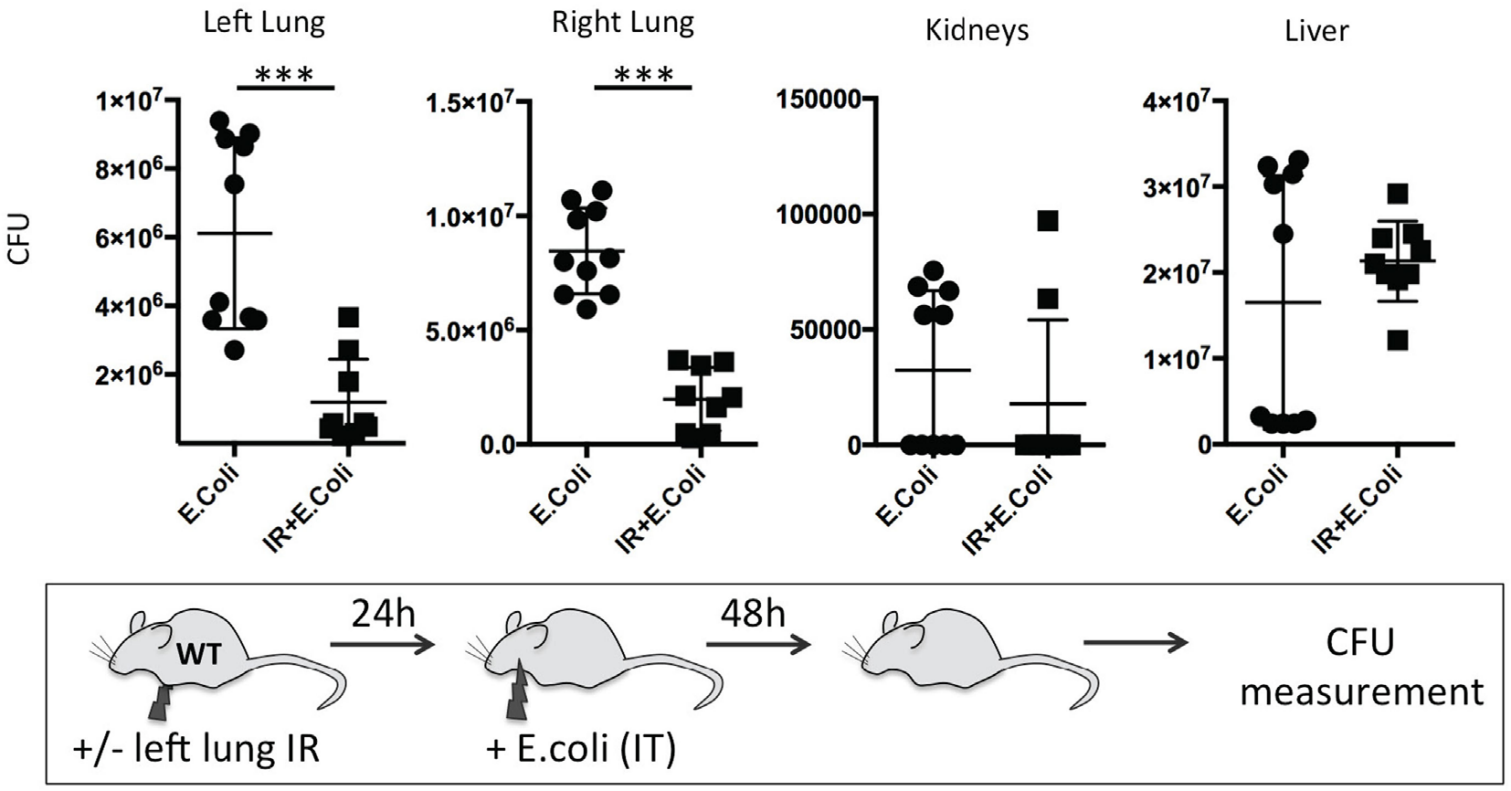
Beneficial effect of lung ischemia–reperfusion (IR) on controlling bacterial pneumonia *in vivo* persists for 24 h after reperfusion. Wild-type mice either received lung IR or not, followed 24 h later with intratracheal (IT) instillation of *Escherichia coli*. Approximately 48 h later, plasma and selected organs were collected, homogenized, and colony-forming unit (CFU) measured. Each data point represents the CFU obtained from the respective sample from a single mouse. Data in the figures are expressed as mean ± SD. Data from *in vivo* studies comparing two conditions were analyzed using two-tailed nonparametric Mann–Whitney analyses.

Finally, we sought to determine the duration of this beneficial effect of lung IR inflammation. We speculated that after a certain period, a second phase of immune paralysis or immunosuppression may create ideal conditions for a pathogenic infection to flourish. We found that even at 24 h following reperfusion, the beneficial effects, though diminished, were still observable in lungs of infected wild-type mice with 10-fold lower bacterial burden compared to mice that did not receive preceding lung IR (Figure 12). Thus, we conclude that the inflammatory process initiated by lung IR is capable of limiting the establishment or spread of an experimental bacterial pneumonia involving *E. coli* even 1 day after reperfusion.

## Discussion

Our study indicates that lung IR inflammation is mediated by the NLRP3 inflammasome and IL-1β and may have beneficial effects to the host in the context of superimposed bacterial lung infection. These conclusions are based on the following experimental evidence. First, we demonstrated that in the absence of all inflammasome subtypes, and specifically the NLRP3 subtype, the inflammation generated following lung IR was significantly attenuated. Subsequently, we provided strong evidence to demonstrate a requirement for caspase 1 activation and IL-1β activity in this inflammatory process while showing that AT2 epithelial cells were likely the primary IL-1β expressing cell and that downstream IL-1R signaling was also required. Next, we observed that while lung IR invoked an inflammatory response involving neutrophil trafficking to the injured lung, these neutrophils were “dormant,” i.e., not activated, but were still capable of activation. Finally, while investigating the possible beneficial effects of this sterile lung IR inflammatory response, we determined that the presence of NLRP3-dependent lung IR inflammation was able to reduce the bacterial burden created by an experimental pneumonia. Furthermore, this beneficial effect persisted for 24 h following reperfusion.

The existence of multiple neutrophil subsets was first proposed by Tsuda et al. and work from the groups of Paul Kubes, Andrew Luster, and others support a model in which multiple waves of neutrophils are attracted to sites of injury by specific chemokine repertoires released at different times (30, 47, 48). Implicit in their findings that there are different subsets of neutrophils with different roles is the likelihood that neutrophils may possess different levels of activity or activation. Our data are consistent with the concept that neutrophils recruited to lung IR injury have not yet converted to their full effector state and may instead serve the purpose of tissue surveillance following injury to a vital organ system. These dormant but ready neutrophils may convert to an activated effector state after an encounter with a foreign or pathogenic threat. These results are in line with the findings of Matthay et al., who in 1989 observed that leukotriene B4 instillation in human volunteer lungs resulted in the recruitment of non-damaging neutrophils (49). Furthermore, Carles et al. reported worsened lung injury from pseudomonal pneumonia when sterile inflammation was dampened through the induction of a heat-shock response (50). These reports are consistent with our findings that inflammation generated by lung IR can augment the host defensive response against bacterial infection.

NOD-, LRR-, and pyrin domain-containing 3 (NLRP3) is widely recognized as having a central role in various sterile inflammatory processes and conditions, including trauma/hemorrhage, gout, tissue necrosis, Alzheimer’s, atherosclerosis, and renal IR (4, 14, 27, 51–53). Furthermore, the NLRP3 inflammasome has been implicated in isolated non-lung models of IR, including myocardial, hepatic, cerebrovascular, and renal IR [(14, 18, 27, 54) and reviewed in Ref. (10)]. Most notably, an NLRP3 inhibitor has been shown to improve injury in a myocardial IR model in mice (55). Finally, IL-1 based therapies are in development and use, including some that target the NLRP3 inflammasome demonstrating the functional importance of IL-1β and the regulation of its production in a broad range of inflammatory processes and diseases (56, 57). However, none of these studies that focused on NLPR3 or on lung injury addressed the issue of lung IR (51, 58). Our findings that the NLPR3 inflammasome and downstream IL-1β signaling are involved in ventilated lung IR injury may not be altogether unexpected given published reports on its role in sterile inflammation. However, ours is the first to report that this pathway is critical in lung IR, especially in the context of coexisting bacterial infection.

The identity of a specialized cell type or types that regulate the processing and release of IL-1β within the spatial cellular organization of the lung *in vivo* is unknown to the best of our knowledge. Most if not all reports identifying cells that produce IL-1β have been focused on *in vitro* differentiated or cultured cell populations or *ex vivo* cells after stimulation (usually with LPS). Our data clearly demonstrates that within the *in vivo* cellular context of the unperturbed lung, IL-1β and surfactant C mRNA signals colocalize and can overlap, leading us to conclude that the AT2 epithelial cell produces IL-1β in the lung. Cells expressing markers for other airway-associated cell types such as AT1 cells or AMs, do not colocalize with IL-1β. We speculate that these IL-1β producing cells form part of the early response to sterile injury and likely do so in conjunction with adjacent AMs, associated fibroblast subtypes, and nearby endothelial cells. Identification of these cells and the specific cellular context for the production and sensing of IL-β as well as downstream products such as IL-6 and CXCL- and CCL-family chemokines is an important advance in understanding the intracellular and intercellular regulation and chronology of this sterile inflammatory process. Furthermore, we found that not all AT2 cells appear to produce IL-1β message both at baseline and after LPS or IR challenge. It is intriguing to speculate whether this production is a stochastic process or instead an indication that different subsets of AT2 cells exist with different roles to play in lung immune biology. Lung epithelial cells are ideally situated to play important roles in regulating diverse lung functions and may do so through the activity of specific mediators they produce [reviewed in Ref. (59)]. In fact, Evans et al. have reported a phenomenon of inducible resistance to bacterial infection in lung epithelium exposed to PAMPs, with evidence for a role for direct killing as well as resistance to pathogen dissemination from the lung [reviewed in Ref. (60)]. Our study does not address the differential contributions of recruited neutrophils and enhanced resistance of lung epithelium to the containment of the experimental bacterial pneumonia we created following lung IR. However, the source of residual resistance to infection present at 24 h following IR may originate from lung endothelium and this observed extended resistance even after the resolution of neutrophilia (Figure 12) is consistent with other reports in the literature (61, 62).

Though our study suggests that lung IR injury creates inflammation that provides a beneficial host effect against bacterial pneumonia, IR inflammation in the lung and in general has been classically viewed as a maladaptive response. In fact, multiple studies have focused on the inhibition of “detrimental” lung IR inflammation to help improve or preserve organ function usually in the context of lung transplantation (63–66). Sterile injury is believed to initiate an inflammatory response whose principal goal is “restoration of physiological homeostasis through tissue repair” with accompanying fibrosis and scarring (47, 67). In contrast, we demonstrated that lung IR inflammation may not to be in and of itself damaging and not cause observable fibrosis or scarring (34). To the best of our knowledge, ours is the first study to examine the consequences and possible benefits of lung IR inflammation on the host response to bacterial infection.

Understanding critical signaling pathways that control lung IR inflammation, characterizing the neutrophils recruited and studying the interaction between inflammation and infection moves the field forward by providing a clear example of a beneficial role for inflammasome-mediated sterile lung inflammation. Furthermore, these results call in to question the universal value of therapies involving inflammation suppression. Perhaps instead, the focus ought to be on identifying clinical circumstances when inflammation suppression may be clearly beneficial (e.g., lung transplantation) and those in which the benefit is unclear or detrimental (e.g., sick ICU patients requiring mechanical ventilation). Since timing of intervention may be just as crucial as the nature of the intervention, new technologies to determine each patient’s unique inflammatory state over the course of their illness need to be developed and validated. With this information we may be able to risk stratify patients into groups requiring different levels of specialized care and types of medical management. For example, repeated sampling of circulating immune cells in blood and AMs in bronchoalveolar lavage fluid could allow for the assessment of the current status of lung and systemic immune activation. Examining active signaling pathways and response to *in vitro* toxin/pathogen challenge in these cells, can provide valuable insight into each individual’s systemic and lung immunity and possibly guide therapy (68, 69). By demonstrating the beneficial aspects of lung IR inflammation in clearing bacterial infections, we favor caution in use of therapeutics that may non-specifically suppress this inflammation, lest the therapy cause more harm than good. This is especially relevant given our recently reported findings that the gut microbiome is required to prime lung sterile immune responses and treatment with antibiotics blunted the lung IR inflammatory response (34).

Our study has some limitations. The conclusions we reach may only be applicable to specific scenarios involving ventilated lung IR and not to lung IR that involves hypoxia, i.e., the contribution of the second “hit” of hypoxia is not addressed here. IL-1α can also play a role in sterile inflammation (70) and our experiments do not formally exclude this possibility. While we show a role for the NLRP3 inflammasome, we cannot exclude the participation or relative importance of other inflammasome subsets nor can we exclude an IL-1β-independent role for NLRP3 (71). Our bacterial pneumonia experiments employ gram negative bacteria, and these results may not apply to gram positive, viral or fungal pneumonia. Comparing these studies to ones involving *Staphylococcus aureus* pneumonia would help address this issue. Additionally, we have yet to define the role of local and systemic inflammation generated by left lung IR in limiting bacterial dissemination from the lungs versus reducing bacterial burden in the seeded organs after the infection has spread. The contribution of NLRP3 to the beneficial effects of IR inflammation therefore may not be limited solely to controlling early IL-1β production or recruitment of dormant neutrophils following injury. NLRP3 may also play a direct role in other cell types, such as in the lung epithelium as was discussed earlier, by preventing bacterial dissemination or promoting bacterial clearance. Our future experiments will seek to delineate the vulnerable period of host immune suppression during which weakened host defenses are unable to respond adequately to infection. Finally, we only used male mice in our study primarily because traumatic injury with hemorrhage that can commonly lead to lung IR and ALI in patients occurs disproportionately in the human male population. Consequently, our data cannot exclude the possibility that lung IR responses in females could be markedly different.

**Figure 13:**
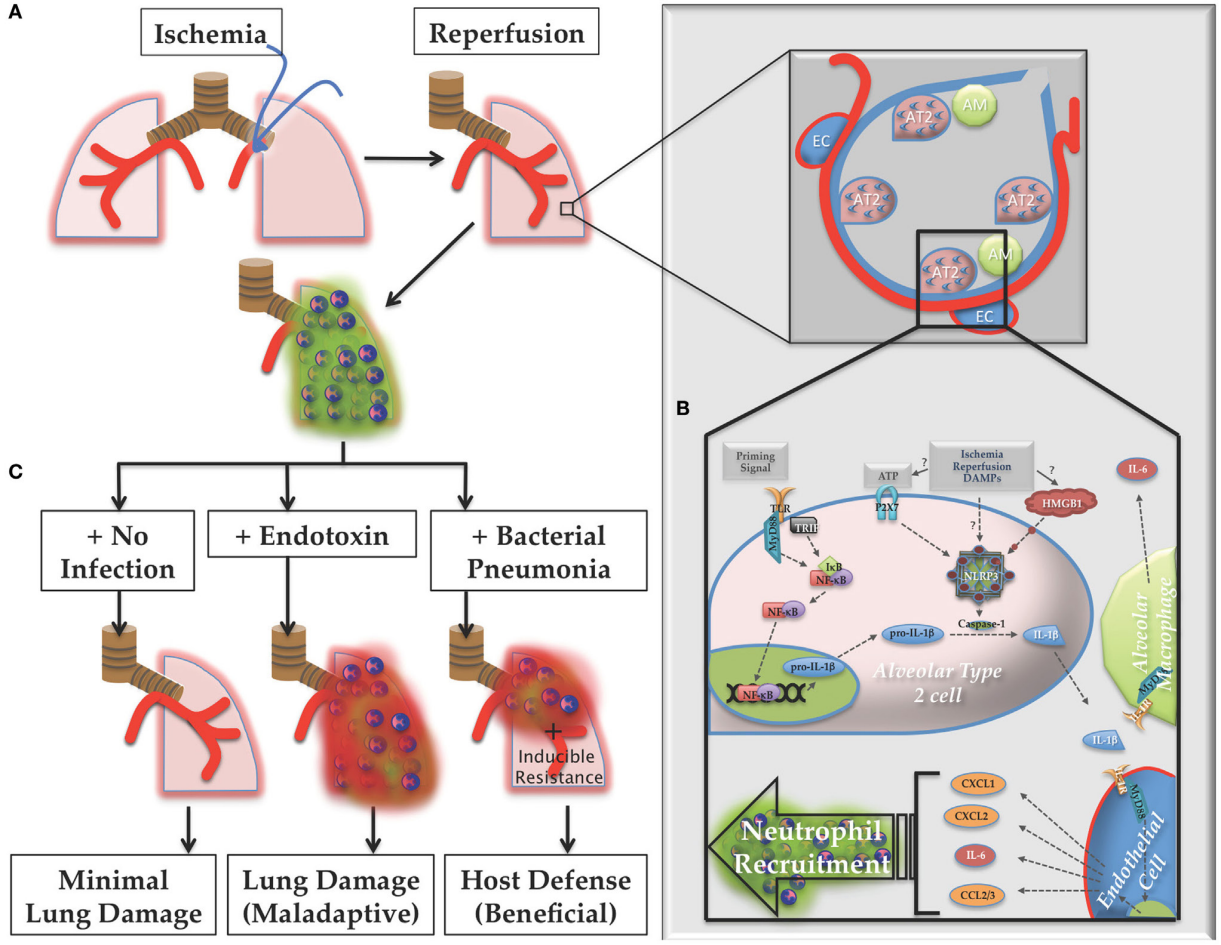
Proposed model of lung ischemia–reperfusion (IR) biology. **(A)** Following ischemia and reperfusion, neutrophils are recruited to the lungs (designated as green dormant neutrophils). **(B)** Within the alveoli the recognition of the damage markers generated by IR by alveolar lining cells, likely alveolar type 2 (AT2) cells, result in NOD-, LRR-, and pyrin domain-containing 3 (NLRP3) inflammasome assembly, caspase processing, and interleukin (IL)-1β processing and active IL-1β release. Nearby endothelial cells respond to this IL-1β and produce IL-6 and neutrophil recruiting chemokines, such as CXCL1, CXCL2, and CCL2/3, resulting in the dormant neutrophil recruitment as well as potentially inducing resistance phenotypes in lung parenchymal cells, such as lung epithelium. **(C)** If the post-IR lung environment remains sterile without further injury, minimal lung damage ensues. However, if the lung encounters pathogenic toxin (LPS, for example), or live pathogen, the dormant neutrophils can convert to activated neutrophils (designated as red activated neutrophils), which along with an activated lung epithelium can contribute to either lung damage (maladaptive immune pathology) or host defense against infection (beneficial immune response), which may include neutrophil activation and other mechanisms of induced resistance, the details of which are yet to be worked out.

In summary, in this study, we established a key *in vivo* role for IL-1β in lung IR inflammation through the activity of the NLRP3 inflammasome. IL-1β release and downstream signaling resulted in the recruitment of neutrophils to the injured lung, which we describe as “dormant” due to their capacity to remain in a suspended state of preactivation. Our finding that AT2 cells are likely the primary IL-1β producing cells within the lung *in vivo* has not been previously reported in the literature. Finally, we demonstrated that IR inflammation enhanced the host defense against a concurrent bacterial lung infection and did so in an inflammasome-dependent manner. The likely beneficial value of preemptive dormant neutrophil recruitment after sterile lung injury, and the identification of the IL-1β producing cell significantly advances our understanding of the pathophysiology of lung inflammation and lung biology in general and is summarized in the schematic model shown in Figure 13. Overall, given the limited targeted medical therapies currently available to treat severely injured and ill hospitalized patients, the need to understand fundamental aspects of lung immunity, and ways to harness it appropriately, remains vitally important.

## Ethics Statement

This study was carried out in accordance with the recommendations of the Institutional Animal Care and Use Committee at the University of California, San Francisco and the corresponding protocol was approved and has been active for the duration of the animal studies presented (Protocol# AN152943).

## Author Contributions

XT performed most in vitro and some *in vivo* experiments and analyzed some data. SH assisted in bacterial pneumonia experiments and analyzed data. AJC assisted in dihydrorhodamine studies and analyzed data. EL and KPF assisted with in vivo imaging experiments and helped analyze data JH assisted with experimental design, editing of manuscript. AP conceived and designed experiments, performed most *in vivo* experiments, wrote, and edited manuscript.

## Conflict of Interest Statement

AC is employed by Amgen, Inc. EL is employed by Murigenics, Inc. KF is employed by PerkinElmer. The remaining authors have no competing interests to declare.
