# Supplementary figures and images for: NLRP3 Inflammasome Mediates Dormant Neutrophil Recruitment following Sterile Lung Injury and Protects against Subsequent Bacterial Pneumonia in Mice
Source: Front Immunol. 2017 Oct 31;8:1337. doi: 10.3389/fimmu.2017.01337 (PMC5671513; doi:10.3389/fimmu.2017.01337)

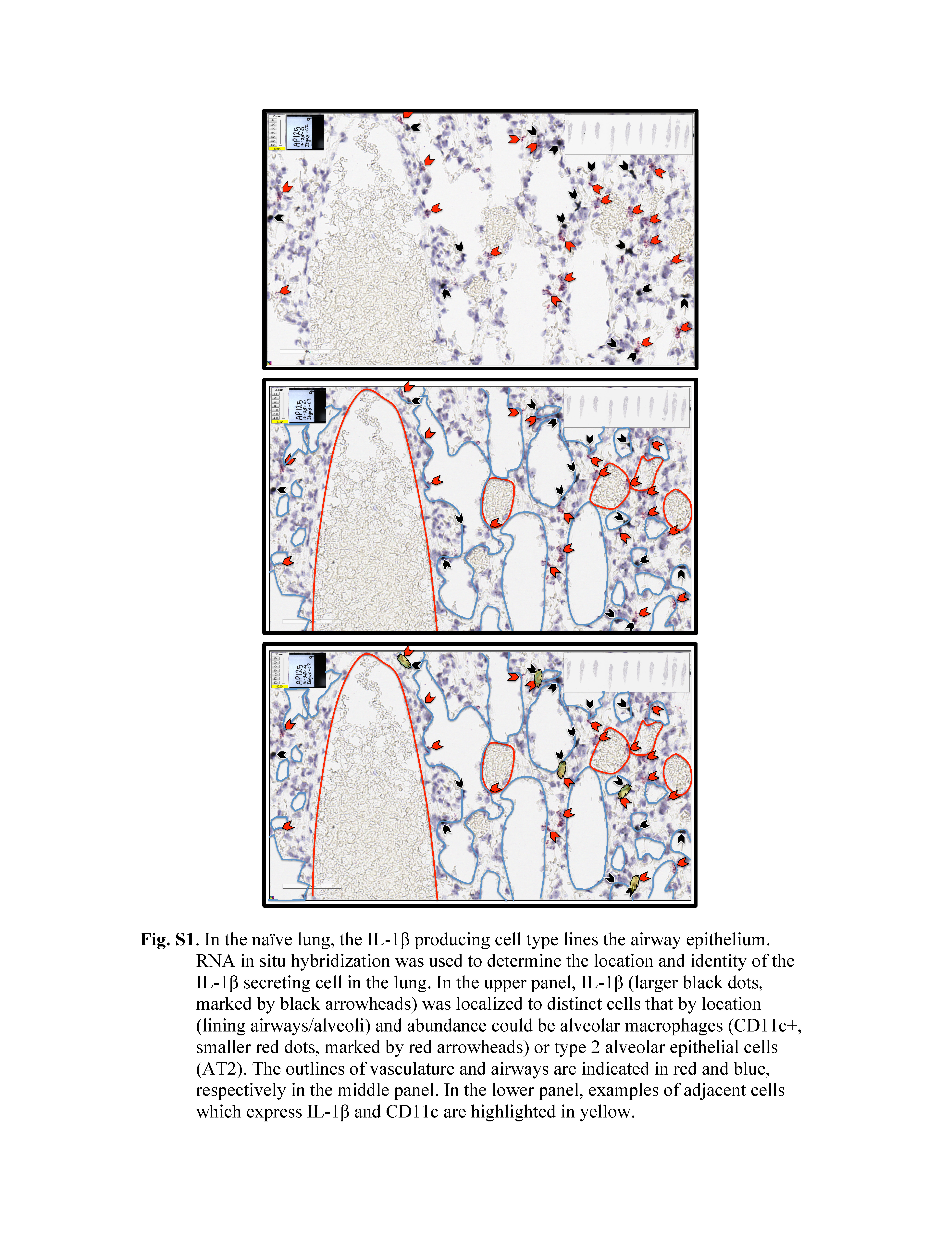

Supplement: Supplementary file 1 [file image_1.jpeg]

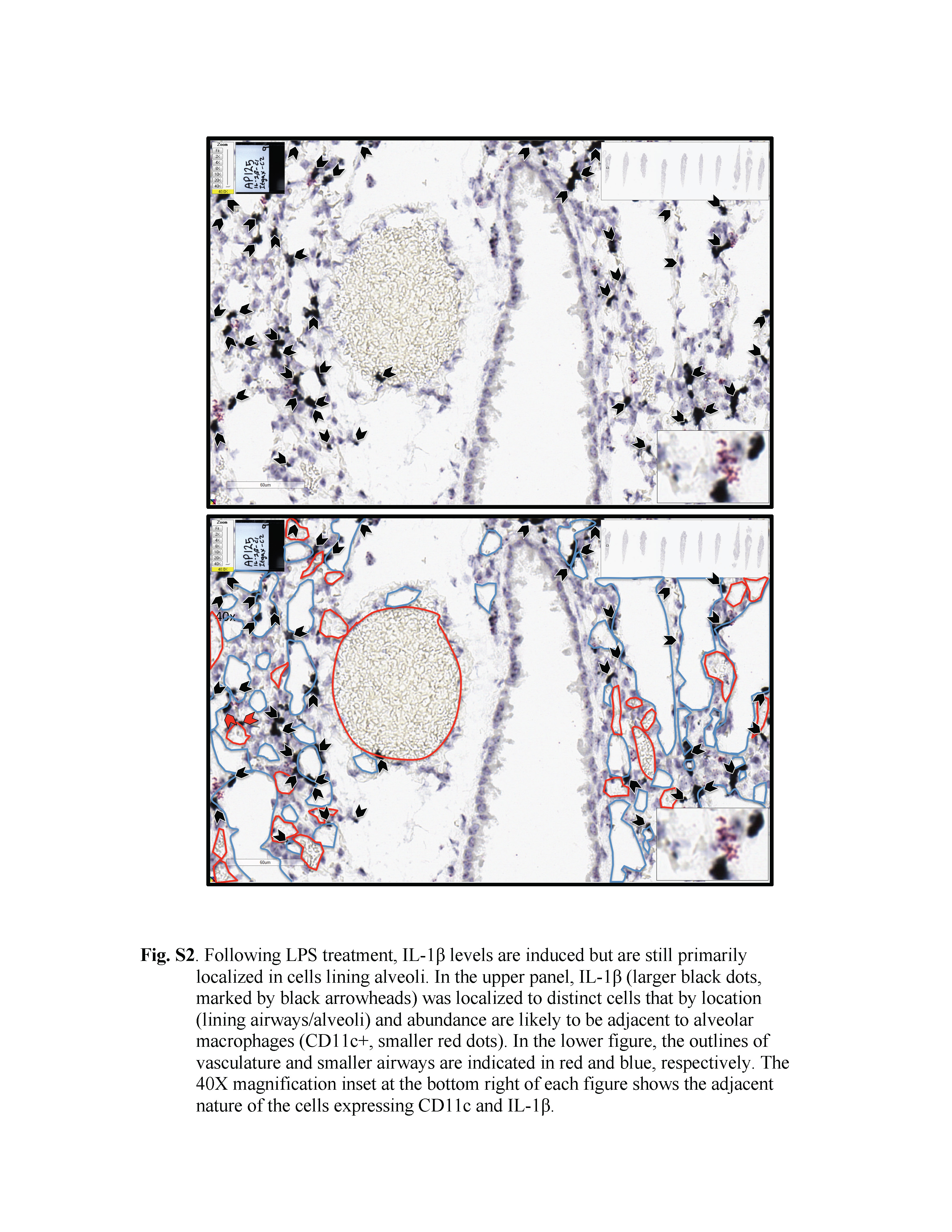

Supplement: Supplementary file 2 [file image_2.jpeg]

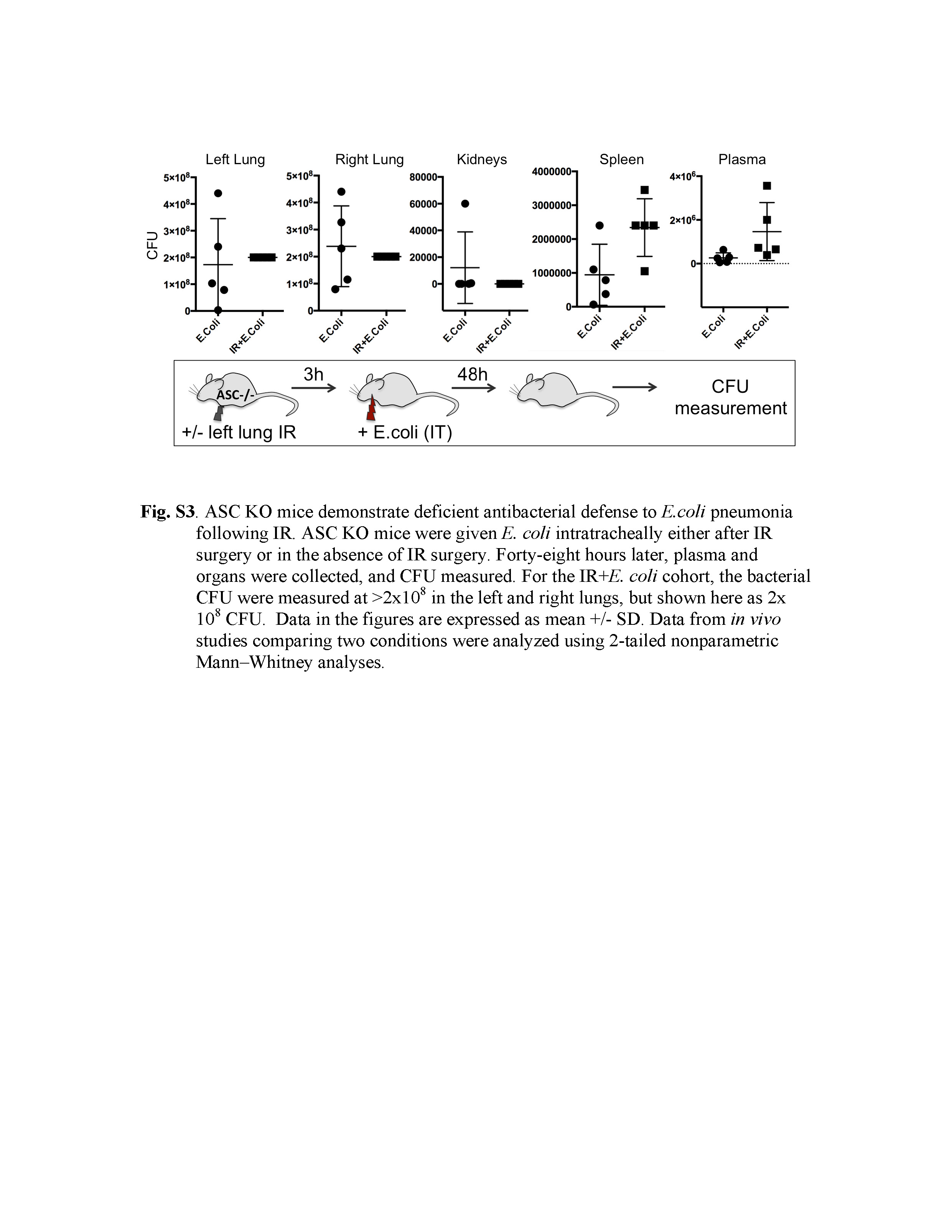

Supplement: Supplementary file 3 [file image_3.jpeg]
